# Role of the trophoblastic BMP7-TGM2 axis in the pathogenesis and prevention of preeclampsia

**DOI:** 10.1038/s41419-026-09111-3

**Published:** 2026-07-11

**Authors:** Qiaoqiao Ding, Mingxi Liu, Binbin Zhao, Yaxin Su, Ying Zhong, Cameron Klein, Junhao Yan, Peter C. K. Leung, Zi-Jiang Chen, Daimin Wei, Yan Li

**Affiliations:** 1 State Key Laboratory of Reproductive Medicine and Offspring Health, Center for Reproductive Medicine, Institute of Women, Children and Reproductive Health, Shandong University, Jinan, China; 2 Medical Integration and Practice Center, Shandong University, Jinan, China; 3 Cardiovascular Research Center of the General Medical Services, Massachusetts General Hospital, Boston, MA USA; 4 Department of Obstetrics and Gynecology, BC Children’s Hospital Research Institute, University of British Columbia, Vancouver, BC Canada

**Keywords:** Pre-eclampsia, Differentiation, Cell invasion, Translational research, Reproductive disorders

## Abstract

Preeclampsia (PE) is a major hypertensive disorder of pregnancy and a leading cause of maternal and perinatal mortality and morbidity worldwide. Aberrant placental development, characterized by shallow trophoblast invasion and incomplete remodeling of maternal spiral arteries, remains central to PE pathogenesis. In PE, tissue transglutaminase (TGM2) is dysregulated in the placenta, contributing to vascular dysfunction, inflammation, and impaired trophoblast differentiation, yet the upstream regulatory signals remain unknown. Bone morphogenetic protein 7 (BMP7) is abundantly expressed at the maternal-fetal interface and implicated in vascular homeostasis, but its role in regulating trophoblast function, especially related to PE pathogenesis, remains undefined. Here, single-cell transcriptomic profiling of placental tissues revealed coordinated downregulation of BMP7 and upregulation of TGM2 in PE relative to normotensive controls. Functional assays in immortalized and primary human trophoblasts demonstrated that BMP7 promotes trophoblast migration and invasion by suppressing TGM2 expression through the ALK2/3-SMAD1/5/9-SMAD4 pathway. Trophoblast organoid models further demonstrated the anti-invasive role of TGM2 in trophoblast differentiation during early placentation. In vivo, an adenovirus-expressing sFlt-1 (Ad sFlt-1)-induced PE mouse model showed increased placental *Tgm2* expression, while recombinant BMP7 administration ameliorated PE-like symptoms. Although the protective effects of BMP7 in vivo are likely mediated by mechanisms beyond TGM2 alone, together these findings identify the BMP7-TGM2 axis as an important trophoblast-regulatory mechanism in PE and support BMP7 as a potential therapeutic candidate.

**Figure:**
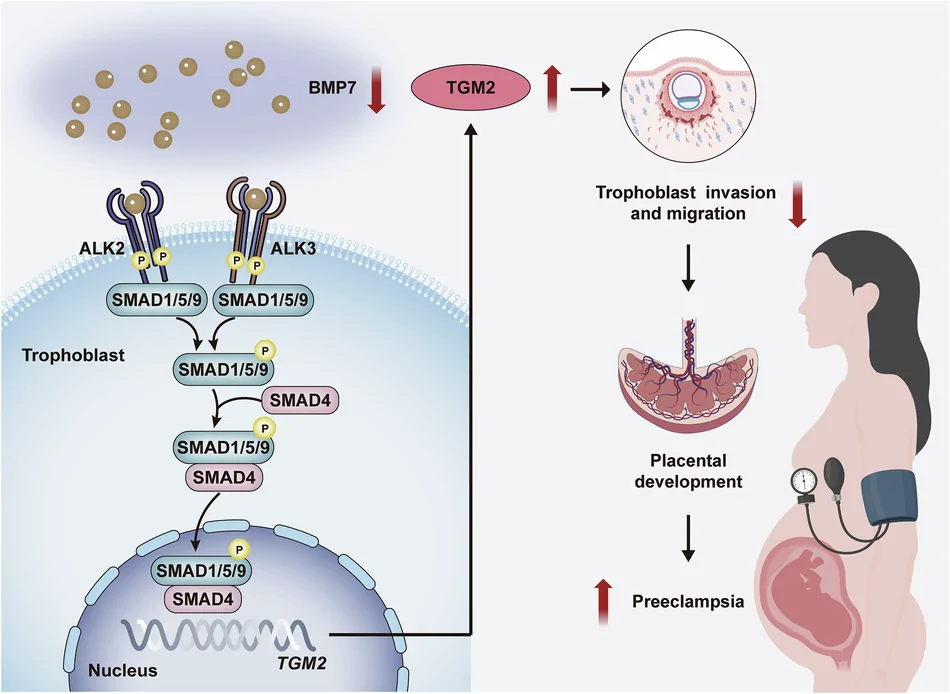

## Introduction

Preeclampsia (PE), a pregnancy-specific hypertensive disorder affecting 3–5% of pregnancies, accounts for over 42,000 maternal deaths annually [1, 2]. Defined by new-onset hypertension and proteinuria or end-organ dysfunction after 20 weeks of gestation, PE is widely regarded as a placental disorder, as symptoms typically resolve following placental delivery [1, 3]. Although its clinical features have been well characterized, the molecular mechanisms that initiate and propagate PE remain poorly understood, limiting effective prevention and treatment strategies beyond delivery.

Human hemochorial placentation is characterized by deep trophoblast invasion into the maternal decidua basalis and myometrium, a process tightly regulated by interactions between trophoblasts and maternal decidua cells [4]. During normal placental development, a subset of villous cytotrophoblasts (VCTs) differentiates into extravillous trophoblasts (EVTs), acquiring invasive capacity essential for endometrial remodelling [5, 6]. Defective trophoblast invasion impairs spiral artery remodeling, leading to persistently narrow maternal vessels and placental hypoperfusion that causes intermittent blood flow and oxidative stress [7]. The ensuing ischemia-reperfusion injury induces cellular oxidative and endoplasmic reticulum stress, which in turn triggers the release of antiangiogenic and pro-inflammatory factors into the maternal circulation, driving endothelial dysfunction and systemic inflammation [1, 7]. Adequate trophoblast invasion is precisely modulated by a plethora of regulating factors, including hypoxia, growth factors, adhesion molecules, and cytokines [8]. Among these, the transforming growth factor-β (TGF-β) superfamily plays a vital role in the spatiotemporal regulation of trophoblast invasion, acting both synergistically and antagonistically [9]. While several TGF-β superfamily members have been extensively studied, the functions of others remain incompletely understood and merit further investigation.

Bone morphogenetic protein 7 (BMP7), a member of the TGF-β superfamily, is an FDA-approved therapeutic for treating bone fractures and a key regulator of diverse developmental processes [10]. Within the reproductive system, BMP7 is upregulated in the endometrium during the receptive phase and is essential for implantation and placental development, as evidenced by placental defects in *Bmp7* conditional knockout mice [11, 12]. Although BMP7 has been shown to promote endothelial proliferation and angiogenic gene expression in trophoblasts, its precise role in trophoblast differentiation and invasion remains unclear. Beyond reproduction, BMP7 has shown therapeutic potential in cardiovascular and metabolic diseases by promoting M2 macrophage polarization and suppressing inflammation [13, 14]. Given the central role of vascular dysfunction and inflammation in PE pathogenesis [15], these findings prompted us to investigate the role of BMP7 in trophoblast regulation and PE development, and to explore its therapeutic potential.

Tissue transglutaminase (TGM2) is the most widely expressed member of the transglutaminase enzyme family, catalyzing post-translational modification of proteins by forming ε-(γ-glutamyl)-lysine isopeptide bonds [16]. Unlike other transglutaminases, TGM2 exhibits broad tissue distribution, with expression in nearly all mammalian cell types [16]. TGM2 participates in numerous biological processes, including cell death, growth, differentiation, and tissue repair, and plays a significant role in the onset and progression of various diseases [16, 17]. Despite its high expression in the placenta, the function of TGM2 in trophoblast activity remains unclear [18]. TGM2 expression is induced by inflammatory cytokines and hypoxia-inducible factor 1 (HIF-1), both of which are elevated in preeclamptic placentas [16, 17]. Notably, placental TGM2 levels are chronically elevated in women with PE [18]. The isopeptide modification mediated by TGM2 modifies the angiotensin receptor type 1 (AT1), stabilizing the receptor by preventing its ubiquitination-dependent degradation, a mechanism implicated in the pathogenesis of PE [18]. While the trophoblast invasion inhibitors TGF-β1/2 have been shown to upregulate TGM2 [19], it remains unknown whether BMP7, a TGF-β superfamily member with opposing vascular and immunomodulatory effects, modulates TGM2 expression in trophoblasts.

Here, analysis of single-cell RNA sequencing (scRNA-seq) data from the first-trimester maternal-fetal interface revealed abundant BMP7 and TGM2 expression in villous trophoblasts. In preeclamptic placentas, scRNA-seq showed reduced BMP7 and elevated TGM2 expression compared to healthy controls, findings that were further validated in additional clinical samples. In vitro, BMP7 promoted trophoblast migration and invasion by suppressing TGM2 expression via the ALK2/3-SMAD1/5/9-SMAD4 signaling pathway. Ex vivo, TGM2 restricted trophoblast differentiation and invasion in human trophoblast organoids. In vivo, placental *Tgm2* levels were elevated in an Ad sFlt-1-induced PE mouse model, recapitulating the findings in human preeclamptic placentas, whereas recombinant BMP7 administration restored placental efficiency and ameliorated PE-like symptoms. Together, these findings identify a dysregulated trophoblastic BMP7-TGM2 axis as an important mechanism underlying impaired trophoblast function in PE and support BMP7 as a potential therapeutic candidate.

## Materials and methods

### Ethics approval for human sample studies

The use of human samples in this study was approved by the Ethics Committee of the Center for Reproductive Medicine of Shandong University (Ethical Review Approval No. 50, 2022). All procedures were conducted in accordance with the Declaration of Helsinki. Written informed consent was obtained from all participants prior to sample collection.

### Clinical villous and placental sample collection

First-trimester villous tissues were obtained from normal pregnancies following voluntary artificial abortion at the Second Hospital of Shandong University. A portion of the villous sample was immediately processed for primary human trophoblast cells isolation and culture and trophoblast organoid culture, while the remaining tissue was fixed in 10% (v/v) formalin and embedded in paraffin for subsequent immunostaining.

Third-trimester placental tissues and serum samples were collected from normal pregnant women and those diagnosed with PE from the Second Hospital of Shandong University. The definition of PE followed the guidelines set forth by the American College of Obstetricians and Gynecologists [1, 3]. Fresh human placentas were obtained immediately after delivery from uncomplicated pregnancies. To isolate pure placental villous tissues, the amnion, chorionic plate, and maternal decidua were carefully removed. Villous core tissues were dissected from the middle layer of the cotyledons, strictly avoiding macroscopic blood vessels, calcified regions, and infarcted areas. To minimize red blood cell contamination, the tissues were thoroughly washed with ice-cold PBS. Portions of each tissue sample were then fixed and embedded in paraffin for immunostaining, while the remaining samples were immediately snap-frozen in liquid nitrogen for subsequent RNA and protein extraction.

### Animal studies

The animal experiments in this study were approved by the Ethics Committee of the Center for Reproductive Medicine of Shandong University (Ethical Review Approval No. 51, 2022). CD-1 mice were purchased from Beijing Vital River Laboratory Animal Technology Co., Ltd. All the mice were housed individually under controlled conditions with a 12-h light/dark cycle at 23 ± 3°C and 55 ± 15% humidity, with unrestricted access to water and food. All procedures involving animals were conducted in compliance with the “Guide for the Care and Use of Laboratory Animals” published by the National Institutes of Health [20].

A mouse model of PE was established by administering adenovirus carrying truncated mouse sFlt–1 (10^9^ plaque-forming units in 100 µl, Ad sFlt-1 group) through the tail vein injection on gestation day (GD) 8 [21]. A control group received adenovirus carrying the murine immunoglobulin G2α Fc fragment (10^9^ plaque-forming units in 100 μL, Ad Fc group) [21]. All adenoviruses were prepared by GeneChem (Shanghai, China).

For mating, 8-week-old female mice were paired with 10-week-old male mice at a 2:1 ratio overnight. The presence of a vaginal copulation plug the following morning was designated as GD0. Pregnant mice were then randomly assigned to one of four groups: Ad Fc+saline group, Ad Fc+BMP7 group, Ad sFlt-1+saline group, and Ad sFlt-1 + BMP7 group (*n* = 5 per group) [21–23]. Systolic blood pressure (SBP) was noninvasively measured every 2‒3 days from GD7 to GD18 using a tail-cuff method (BP-2010; Softron, Tokyo, Japan).

From GD10 to GD13, pregnant mice received daily injections of recombinant BMP7 protein (10 μg/kg/day) (Proteintech, HZ-1229) in 100 µL of saline solution, or equivalent volume of saline as a control, via tail vein injection. To pharmacologically inhibit TGM2, the transglutaminase inhibitor cystamine dihydrochloride (cystamine diHCl; HY-W020050, MedChemExpress, Monmouth Junction, NJ, USA) was utilized. A separate cohort of pregnant mice was allocated into three groups (*n* = 3 per group): Ad Fc + saline, Ad sFlt-1 + saline, and Ad sFlt-1 + cystamine diHCl. From GD10 to GD17, mice in the treatment group received daily intraperitoneal injections of cystamine diHCl (50 mg/kg/day, dissolved in sterile saline), while control animals received an equivalent volume of saline. Systolic blood pressure was monitored as described above. At GD18, fetal weight, placental weight, and placental efficiency were evaluated. All animals were monitored daily for general health throughout the treatment period.

All pregnancies were terminated on GD18 under deep anesthesia, following blood collection from the orbital sinus. Serum was isolated by centrifugation at 3000×*g* for 10 min and stored at −80 °C for subsequent analysis. Fetal and placental weights were recorded, and the total number of fetuses (alive and non-viable) was noted. Placental and kidney tissues were harvested, with some portions fixed in 10% (v/v) formalin and embedded in paraffin for morphological studies, while the remaining tissues were flash-frozen in liquid nitrogen and stored at −80 °C for further analysis.

### ScRNA-seq data analysis

Published single-cell transcriptomic datasets of the first-trimester maternal-fetal interface and third-trimester placentas were re-analyzed by the Seurat R package (4.0.5) [24–26]. Cells were initially filtered based on the following criteria: for droplet-based data, a gene count exceeding 500 and a mitochondrial gene percentage below 20%; for Smart-seq2 data, a gene count exceeding 1000 and a mitochondrial gene percentage below 20%. To mitigate batch effects caused by background contamination from cell-free RNA, genes associated with ambient RNA expression, including *PAEP*, *HBG1*, *HBA1*, *HBA2*, *HBM*, *AHSP*, and *HBG2*, were removed. After filtering, the gene-barcode matrices from all samples were integrated to eliminate batch effects across different donors. Specifically, 2000 shared highly variable genes were identified using Seurat’s ‘SelectIntegrationFeatures()’ function. Integration anchors were then determined based on these genes through canonical correlation analysis utilizing 90 dimensions with the ‘FindTransferAnchors()’ function. Subsequently, the data were integrated using ‘IntegrateData()’ and scaled with ‘ScaleData()’. For dimension reduction, principal component analysis (PCA) and uniform manifold approximation and projection (UMAP) dimension reduction with 40 principal components were conducted. A nearest-neighbor graph was generated based on the 40 dimensions of the PCA reduction using the ‘FindNeighbors()’ function, followed by clustering with ‘FindClusters()’ function at a resolution of 0.93. Conserved markers for each cluster were identified using the ‘FindAllMarkers ()’ function. Cells were re-annotated according to markers identified in the original publications. Finally, *BMP7* and *TGM2* expression across different placental cell types were visualized using the ‘FeaturePlot()’ and ‘VlnPlot()’ functions.

### Culture of immortalized human trophoblast cell

The HTR**-**8/SVneo simian virus 40 large T antigen-immortalized human trophoblast cell line was obtained from the American Type Culture Collection (ATCC, USA). Cells were cultured in Dulbecco’s modified Eagle’s medium (DMEM)/F-12 (Gibco, Grand Island, NY, USA) supplemented with 10% (v/v) fetal bovine serum (ExCell Bio, Shanghai, China), 100 U/ml penicillin, and 100 μg/ml streptomycin (HyClone, Logan City, UT, USA) in a humidified incubator (5% CO_2_/95% air at 37 °C). The cell culture media were changed every two days. To minimize interference from serum-derived factors, cells were starved in DMEM/F-12 containing 0.1% (v/v) FBS for 24 h prior to recombinant BMP7 (50 ng/mL) treatment.

### Primary human trophoblast cells isolation and culture

Primary human trophoblast cells were isolated from first-trimester chorionic villi (6–9 weeks of gestation) as described previously [27]. Briefly, first-trimester villi tissues were washed with Dulbecco’s modified phosphate-buffered saline (PBS) (HyClone, Logan City, UT, USA), minced into small pieces, and cultured in DMEM/F-12 supplemented with 10% (v/v) FBS, 100 U/ml penicillin, and 100 μg/ml streptomycin. The tissues were cultured at 37 °C in a humidified 5% CO_2_/95% air atmosphere for 3–4 days, and the nonattached pieces were then discarded. Trophoblast cells outgrow villous tissue fragments after further culture and are subsequently separated by differential trypsin digestion from attached fragments. The purity of primary human trophoblast cells was assessed immunocytochemically, and 100% positive staining for cytokeratin 7 (CK7; a trophoblast and epithelial marker) was detected.

### Trophoblast organoid culture and EVT differentiation induction

First-trimester placental villi (6–8 weeks of gestation) were obtained from healthy women undergoing elective termination of pregnancy, with written informed consent and institutional ethical approval. Villous tissues were rinsed in ice-cold PBS to remove blood, mechanically separated from the chorionic membrane, and subjected to sequential enzymatic digestions followed by Percoll density-gradient centrifugation. Cells collected from the 30–50% interface were washed with HBSS, embedded in ice-cold growth factor-reduced Matrigel (Corning, USA; undiluted), and plated as 25–40 μL domes in 24-well plates. After polymerization at 37 °C for 20 min, 500 μL of trophoblast organoid medium (TOM) was added and replaced every 1–2 days. TOM was prepared exactly as described by Sheridan et al. [28].

For extravillous trophoblast (EVT) differentiation, mature organoids maintained in TOM for 3–4 days were switched to EVT differentiation medium (EVT medium) and cultured for a further 5–10 days with medium refreshed every 2 days. The EVT medium was prepared according to the method described by Sheridan et al. [28]. It was derived from TOM by omitting CHIR99021 and FGF2, while supplementing with 0.5% fetal bovine serum and 4% KnockOut Serum Replacement, and retaining both A83-01 and Y-27632 [28].

For experiments involving recombinant TGM2 treatment, mature organoids maintained in TOM for 3–4 days were switched to EVT medium supplemented with recombinant human TGM2 (2.5 μg/mL) or vehicle control for the indicated period. Recombinant human TGM2 was purchased from Zedira (T002, Darmstadt, Germany). According to the manufacturer, this recombinant human tissue transglutaminase is produced in *E. coli* with a molecular weight of 78 kDa and a purity of >90% by SDS-PAGE. The working concentration of 2.5 μg/mL was chosen based on a preliminary dose-response experiment evaluating EVT marker expression.

At the indicated endpoints, organoids were subjected to bright-field imaging, RNA extraction for qPCR and RNA-seq, and immunofluorescence staining for marker verification.

### Gene knockdown and overexpression

For overexpression of TGM2, FLAG-tagged pCMV-TGM2 plasmids (GenePharma, Shanghai, China) were transfected into cells using Lipofectamine 3000 (Invitrogen Life Technologies, Carlsbad, CA) following the manufacturer’s instructions, and an empty pCMV vector was used as the control.

For gene knockdown, cells were transfected with small interfering RNA (siRNA) using Lipofectamine RNAiMAX (Invitrogen Life Technologies, Carlsbad, CA, USA) according to the manufacturer’s protocol. siRNAs targeting human *BMP7, TGM2, ALK3*, *ALK4*, *ALK5*, *SMAD1*, *SMAD5*, *SMAD9*, *SMAD2*, *SMAD3*, *SMAD4*, and control siRNAs were purchased from GenePharma (Shanghai, China), and ON-TARGETplus SMARTpool si-*ALK2* was obtained from Dharmacon (Lafayette, CO). After 48 h of transfection, protein and mRNA were extracted to assess the transfection efficiency via western blot and RT‒qPCR. The siRNA sequences used in this study are listed in Table S1.

### Wound healing assay

A wound healing assay was used to measure cell migration ability in vitro [29]. Following the creation of a scratch, the migration area was photographed every 24 h in five random fields per wound. Migration rates were quantified using ImageJ software (National Institutes of Health, USA).

### Transwell invasion and migration assays

Transwell invasion/migration assays were carried out in 24-well plates with 8-μm pore size Transwell filter inserts (Corning, Inc., Corning, NY, USA). For invasion assays, the inserts were coated with Matrigel (50 μL of 1 mg/mL; Corning, Inc., Corning, NY, USA), while uncoated inserts were used for migration assays, following a previously described protocol [27]. A 200 μL cell suspension with 0.1% FBS was added to the upper compartment of the transwell inserts, while 600 µl of medium supplemented with 10% FBS was added to the lower compartment. After incubation, cells that invaded or migrated through the membrane were stained with 0.1% (w/v) crystal violet solution (Solarbio Science & Technology, Beijing, China) for 15 min. Five random fields for each sample were photographed and counted under a microscope (Olympus, Tokyo, Japan).

### Bulk RNA-seq and data analysis

Cell or tissue samples were sent to Novogene (Beijing, China) for total RNA isolation, cDNA library construction, and sequencing. RNA quality and integrity were assessed using the RNA Nano 6000 Assay Kit on the Bioanalyzer 2100 system (Agilent Technologies, CA, USA). mRNA was purified from total RNA using poly-T oligo-attached magnetic beads and subsequently fragmented with divalent cations at elevated temperatures.

First-strand cDNA was synthesized using random hexamer primers and M-MuLV reverse transcriptase, followed by second-strand cDNA synthesis using DNA polymerase I and dNTPs after RNA degradation by RNaseH. The resulting library fragments were purified using the AMPure XP system (Beckman Coulter, Beverly, USA) and enriched by PCR to generate the final cDNA library after an end repair, the addition of a single “A” base, and adapter ligation.

Library quantification was performed using a Qubit 2.0 fluorometer (Life Technologies, USA), and insert size was confirmed with an Agilent 2100 bioanalyzer (Agilent Technologies, USA). The effective concentration of the library was accurately quantified by RT‒qPCR. After library qualification, libraries were pooled according to their effective concentration and target data output and sequenced on the Illumina NovaSeq 6000 platform. Reference genome and gene model annotation files were downloaded directly from the genome website. Paired-end clean reads were aligned to the reference genome using HISAT2 (v2.0.5) to construct the genome index. Differential expression analysis between two groups was performed using the DESeq2 R package (1.20.0), with the Benjamini and Hochberg method employed to control the false discovery rate. Significantly differentially expressed genes were identified with an adjusted *P* value < 0.05 and a fold change >1.2.

### Hematoxylin and eosin (H&E) staining

Paraffin-embedded human placental tissues and mouse placental and renal tissues were deparaffinized in xylene twice for 5 min each and rehydrated through a graded ethanol series (100%, 95%, 80%, and 70%) for 2 min each. The sections were rinsed in distilled water for 5 min, stained with hematoxylin for 90 s, and washed under running water for 5 min to remove excess dye. Then, the sections were briefly differentiated in 1% acid alcohol and blued in tap water for 1 min, followed by eosin staining for 15 s and a quick rinse in running water. The stained sections were dehydrated in 95% ethanol and absolute ethanol for 1 min each and then cleared in xylene twice for 5 min each. Images were captured using an APERIO VERSA8 microscope (Leica, Wetzlar, Germany).

### Periodic acid-Schiff (PAS) staining

After deparaffinized in xylene and rehydrated through a graded alcohol series, the sections were oxidized in periodic acid solution for 10 min, washed in distilled water, and stained with Schiff for 15 min. After a final rinse in distilled water to remove excess dye, the sections were counterstained with hematoxylin for 2 min. Then, images were captured using an APERIO VERSA8 microscope (Leica, Wetzlar, Germany).

### Immunohistochemistry (IHC) staining

Consecutive serial sections (5 μm thick) were prepared for IHC staining using an enhanced detection kit (Zhongshan Goldenbridge Biotechnology, Beijing, China) following the manufacturer’s protocol. Sections were first deparaffinized in xylene and rehydrated through a graded alcohol series. Following antigen retrieval for 20 min, sections were incubated with endogenous peroxidase blockers for 25 min and then blocked with 10% (w/v) bovine serum albumin (BSA, Solaibio, Beijing, China) containing 0.3% (v/v) Triton X-100 (Thermo Fisher Scientific Inc., MA, USA) for 60 min at room temperature. The sections were subsequently incubated overnight at 4 °C with primary antibodies. A rabbit immunoglobulin G (IgG) isotype control or a mouse IgG isotype control was used as negative controls. The following day, tissue sections were incubated with appropriate secondary antibodies for 60 min at room temperature, followed by staining with a diaminobenzidine (DAB) substrate kit (Vector Laboratories, Burlingame, CA, USA). Images were captured using a microscope (Olympus, Tokyo, Japan) or an Aperio VERSA scanner (Leica Microsystems, Nanterre, France). H&E staining and PAS staining were performed to assess the general morphology of mouse placental and kidney tissues. The areas of three typical layers within the mouse placenta (decidua, junctional zone, and labyrinth; *N* = 5 per group) and the CD34^+^ area within glomerular capillaries and the placental labyrinth (*n* = 20 glomeruli per mouse or *n* = 12 regions of the placental labyrinth per mouse) were measured using Aperio ImageScope software (Leica Biosystems, Nanterre, France). Antibodies used in this study are listed in Table S2.

### Immunofluorescence (IF) staining

For the paraffin-embedded sections, deparaffinization and rehydration were performed as described above. After antigen retrieval and blocking with 10% (w/v) BSA containing 0.1% (v/v) Triton X-100 (Thermo Fisher Scientific Inc., MA, USA) for 60 min, sections were incubated with primary antibodies overnight at 4 °C. For adherent cells, cells were fixed with 4% (w/v) paraformaldehyde for 20 min, permeabilized with 0.3% (v/v) Triton X-100 for 40 min, blocked with 10% (w/v) BSA in PBS for 1 h, and incubated with primary antibody at 4 °C overnight. For trophoblast organoids, organoids were cultured in confocal dishes (Cellvis, D35-20-1-N), fixed with 4% paraformaldehyde for 30 min at room temperature, permeabilized with 0.5% Triton X-100 in PBS for 30 min, and blocked with 5% BSA in PBS for 1 h. Organoids were then incubated with primary antibodies diluted in 5% BSA/PBS containing 0.05% Triton X-100 overnight at 4 °C. Following three 5-min PBS washes, samples were incubated with appropriate secondary antibodies for 1 h at room temperature. Cell nuclei were counterstained with Fluoroshield Mounting Medium containing 4′,6-diamidino-2-phenylindole (DAPI) (Abcam, Cambridge, MA, USA) for 5 min. Immunofluorescence images were obtained using a fluorescence microscope (Olympus, Tokyo, Japan) or an Olympus VS120 Slide Scanner (Olympus, Tokyo, Japan). For quantification, CK7 staining was used to define the trophoblast compartment, and the fluorescence intensity of BMP7 or TGM2 within the CK7-positive trophoblast area was measured using identical acquisition settings across groups. The antibodies used in this study are listed in Table S2.

### Western blot

Cell and tissue extracts were prepared using ice-cold RIPA lysis buffer (Beyotime Institute of Biotechnology, Nantong, China) containing 1% proteinase and phosphatase inhibitor cocktail (Cell Signaling Technology, USA), followed by centrifugation at 14,000×*g* and 4 °C for 15 min. Protein concentrations were quantified using a BCA protein assay kit (Thermo Fisher Scientific Inc., MA, USA). The protein lysates were separated using 4‒20% sodium dodecyl sulfate‒polyacrylamide gel electrophoresis (SDS-PAGE) and transferred to polyvinylidene difluoride (PVDF) membranes (Millipore, Boston, MA, USA). Membranes were blocked with 5% (w/v) fat-free milk in TBST (Tris-buffered saline with 0.1% Tween-20) for 60 min at room temperature with gentle shaking. After blocking, membranes were incubated with primary antibodies overnight at 4 °C. Excess primary antibody was removed by washing with TBST, and membranes were then incubated with appropriate secondary antibodies for 1 h. Visualization was performed using a ChemiDoc MP Imaging System (Bio-Rad, CA, USA) with an enhanced chemiluminescence kit (Millipore, Boston, MA, US), and the results were analyzed using ImageJ software. β-ACTIN served as the loading control for both cell and tissue samples. The antibodies used in this study are listed in Table S2. All uncropped western blots are available in Supplementary material.

### Enzyme-linked immunosorbent assay (ELISA)

Human and mouse serum samples were separated immediately by centrifugation at 1000×*g* for 10 min and stored at −80 °C until analysis. Human BMP7 levels were measured using a Human BMP7 Quantikine ELISA Kit (Proteintech, Wuhan, CN) following the manufacturer’s instructions (intra-assay CV < 7%, inter-assay: CV < 10%). Mouse serum levels of sFlt-1 was quantified using a mouse sVEGFR1/Flt-1 ELISA Kit (Proteintech, Wuhan, CN; intra-assay CV < 6%, inter-assay: CV < 7%). The optical density (OD) of each well was measured at the appropriate wavelength using a Spectra Max Plus 384 (Molecular Devices, Sunnyvale, CA, USA). Standard curves and concentration calculations were performed using ELISACalc software with four-parameter logistic (4-PL) curve fitting.

### EdU incorporation assay

EdU incorporation assay was used to assess cell proliferation according to the manufacturer’s instructions. Briefly, cells were seeded into 96-well plates and incubated with 50 μM EdU (RiboBio, Guangzhou, China) at 37 °C for 2 h, followed by staining with Apollo 567 and Hoechst 33342 (RiboBio, Guangzhou, China). Immunofluorescence micrographs were captured using a fluorescence microscope (Olympus, Tokyo, Japan), and the number of EdU-positive cells was determined using ImageJ software (National Institutes of Health, USA). Each test group included at least three wells, with triplicate microscopic fields counted per well. At least three independent experiments were performed.

### Detection of apoptosis by flow cytometry

To detect apoptosis, cells were washed twice with cold PBS after the removal of the culture supernatant and then digested with 0.25% trypsin. The collected cells were centrifuged at 800 rpm for 5 min, washed with cold PBS, and resuspended in 500 μL of 1× binding buffer. Each sample was supplemented with 5 μL of Annexin V-APC and 10 μL of 7-AAD (MultiSciences Biotech, Hangzhou, China) and incubated in the dark at room temperature for 5 min. All the samples were placed on ice, and apoptosis was analyzed within 1 h using flow cytometry (BD FACSCalibur, BD Biosciences, CA, USA).

### RT-qPCR

Total RNA from tissues and cultured cell lines was extracted using TRIzol reagent (Invitrogen, MA, USA) according to the manufacturer’s instructions. RNA concentration and purity were assessed using a NanoDrop One spectrophotometer (Thermo Fisher Scientific, USA). Total RNA was reverse transcribed into cDNA using the Prime Script RT Reagent Kit with gDNA Eraser (TaKaRa, Kusatsu, Japan). mRNA expression levels of target genes were quantified using TB Green™ Premix Ex Taq™ II (TaKaRa, Kusatsu, Japan) on a LightCycler 480 system (Roche, Penzberg, Germany) and normalized to the expression of the housekeeping gene *ACTB* or *Gapdh* via the 2^−∆∆Ct^ method. Primer specificity was confirmed through dissociation curve analysis of PCR products. Each sample was analyzed in triplicate, with a minimum of three independent experiments performed. Primer sequences used in this study are listed in Table S3.

### Other chemicals and reagents

DMH-1 (S7146) and SB431542 (S1067) were obtained from Selleck Chemicals (Houston, TX, USA). Cystamine diHCl (HY-W020050) was purchased from MedChemExpress (Monmouth Junction, NJ, USA).

### Statistical analysis

Statistical analyses were conducted using GraphPad Prism 9 (GraphPad Software Inc., USA) and SPSS 20.0 (IBM, USA). The Kolmogorov-Smirnov test combined with histograms was used to test the normality of data distribution. For normally distributed data, comparisons between two groups were performed using unpaired two-tailed Student’s *t* tests, and differences among more than two groups were assessed using one-way or two-way analyses of variance (ANOVA), followed by Tukey’s post hoc analysis for multiple comparisons. For non-normally distributed data between two groups or more than two groups, the Mann–Whitney *U* test or Kruskal–Wallis H test were used. Categorical variables were compared using the chi-square test or Fisher’s exact test. A *P* value of <0.05 was considered statistically significant. All experiments were performed at least three times independently.

## Results

### Downregulation of trophoblastic BMP7 in PE placentas

**Fig. 1:**
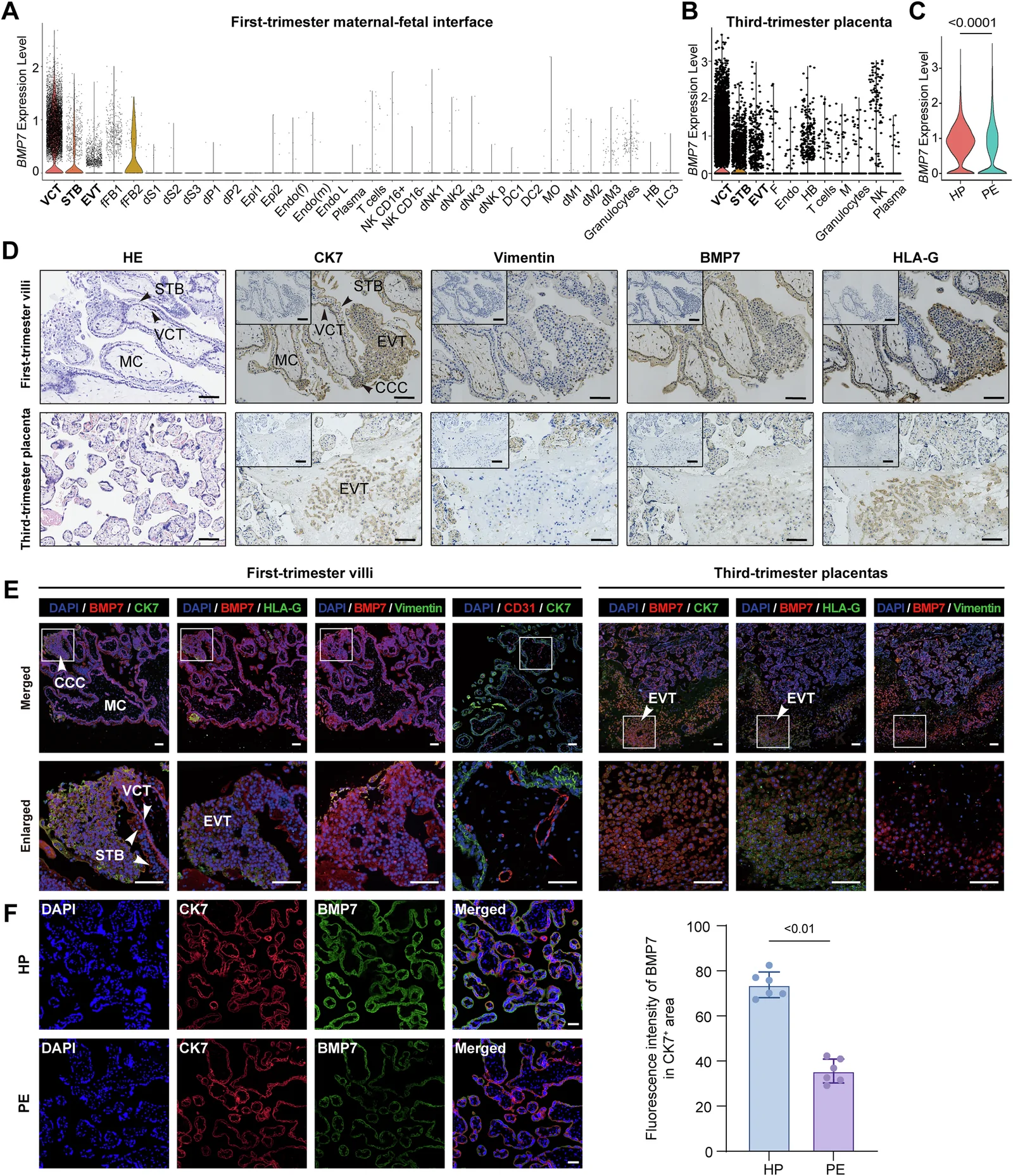
BMP7 expression is downregulated in VCTs from PE placentas. **A** Violin plot displaying the expression of *BMP7* in all captured cell types at the first-trimester maternal-fetal interface. High expression of *BMP7* was detected in VCT, STB, and EVT. **B**, **C** Single-cell transcriptomes of term placentas containing 5 preeclamptic placentas and 5 healthy placentas. Violin plot (**B**) displaying the *BMP7* expression level in all cell types. Violin plot (**C**) displaying the differential expression of *BMP7* in VCT cells from preeclamptic (PE) and healthy pregnancy (HP) placentas. **D** Hematoxylin and eosin (H&E) staining and immunohistochemistry localization of BMP7 in first-trimester villi and third-trimester human placental tissues. CK7 was used as a marker for placental trophoblasts and epithelial cells; HLA-G was used as a marker for EVTs; and vimentin was used as a marker for villous MCs and uterine SCs. Scale bars: 100 μm. **E** Immunofluorescence localization of BMP7 in first-trimester villi and third-trimester human placental tissues. CD31 was used as a marker for endothelial cells. Scale bars: 50 μm. **F** Immunofluorescence staining of CK7 and BMP7 in third-trimester human placental tissues from 6 PE donors and 6 HP donors. Representative images are shown, and the fluorescence intensity of BMP7 was quantified in the CK7-positive trophoblast compartment. Scale bars: 50 μm. Data are presented as the means ± SD and were analyzed by unpaired two-tailed Student’s *t* tests. *P* < 0.05 was considered statistically significant. ns not significant, CCC cytotrophoblast cell column, CK7 cytokeratin-7, d decidual, dM decidual macrophages, dS decidual stromal cells, DC dendritic cells, Endo endothelial cells, Epi epithelial glandular cells, EVT extravillous trophoblast, f fetal, F fibroblasts, GE glandular epithelium, HB Hofbauer cells, HLA-G human leucocyte antigen-G, l lymphatic, ILC3 type 3 innate lymphoid cells, M macrophages, M3 maternal macrophages, MCs mesenchymal cells, MO monocytes, m maternal, NK natural killer cells, SCs stromal cells, STB syncytiotrophoblast, VCT villous cytotrophoblast.

To investigate the potential role of BMP7 in trophoblast function and PE pathogenesis, we first examined cell-type-specific BMP7 expression patterns at the maternal-fetal interface using public single-cell RNA sequencing (scRNA-seq) datasets (E-MTAB-6701, E-MTAB-6678 and GSE173193) [24–26]. These datasets encompass a comprehensive range of cell populations from the first-trimester maternal-fetal interface and third-trimester placentas (Fig. 1A, B). In both gestational stages, villous cytotrophoblast (VCTs) were identified as the primary BMP7-expressing cells (Fig. 1A, B). Differentially expressed gene (DEG) analysis revealed significantly lower *BMP7* expression in VCTs from PE placentas compared with healthy pregnancy (HP) placentas, suggesting a potential role for trophoblastic BMP7 downregulation in PE development (Fig. 1C).

To validate these scRNA-seq findings, we performed immunostaining for BMP7 alongside trophoblast (cytokeratin-7, CK7) and mesenchymal (vimentin) markers using first-trimester villous and third trimester placental tissues. BMP7 was abundantly expressed in trophoblasts across gestational ages, suggesting both autocrine and paracrine roles in trophoblast function (Fig. 1D, E). We further examined the trophoblastic expression levels of BMP7 from six PE patients and six gestational age-matched controls, with the baseline characteristics of participants presented in Table S4. As indicated by the co-localization with CK7, trophoblasts from PE placentas exhibited significantly weaker BMP7 staining than those from HP placentas, consistent with the scRNA-seq findings of trophoblastic BMP7 deficiency in PE patients (Fig. 1F). Simultaneously, we collected placental villous tissues from the PE and HP groups to evaluate differential BMP7 expression at both mRNA and protein levels. Consistent with the immunofluorescence findings, RT-qPCR and western blot analyses of placental villous tissue further confirmed reduced BMP7 expression in PE placental villi (Fig. S1A, B).

Given that BMP7 is a secretory growth factor, we next examined maternal serum samples to assess whether circulating BMP7 levels are dysregulated in PE and evaluate its potential as a circulatory biomarker. However, ELISA assays revealed that BMP7 was undetectable in maternal serum from both PE (*n* = 23) and HP (*n* = 11) groups. The clinical characteristics of the PE patients and HP donors included in these assays are summarized in Table S5. The absence of detectable BMP7 in the maternal circulation suggests that its function is predominantly localized to the placenta, rather than exerting systemic effects on maternal vasculature.

### BMP7 promotes trophoblast migration and invasion

**Fig. 2:**
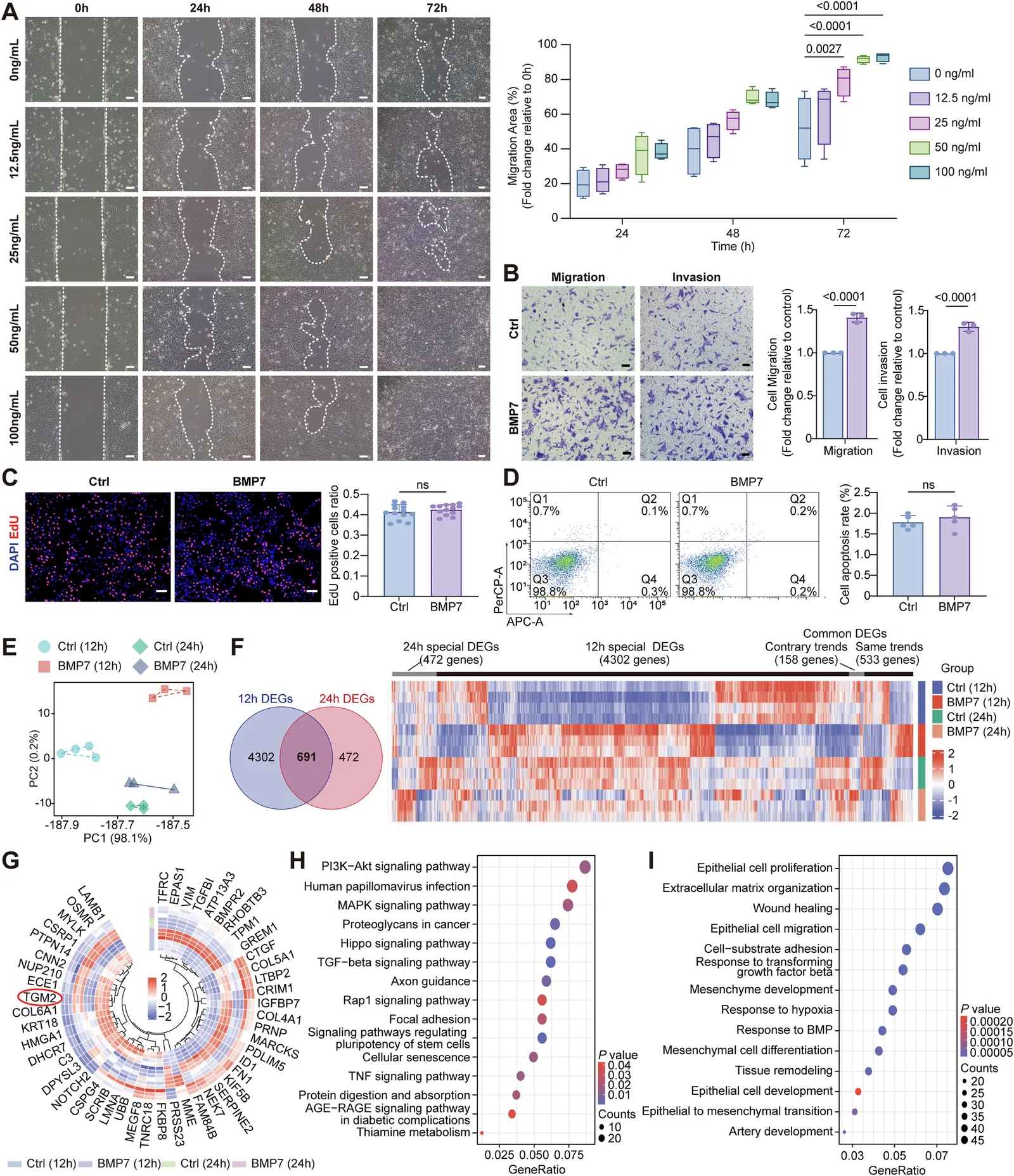
BMP7 promotes invasion and migration of trophoblasts in vitro. **A** HTR-8/SVneo cells were treated with different concentrations of recombinant BMP7 (0–100 ng/mL), and the relative migration area in each group was imaged and quantified every 24 h. Scale bars: 100 μm. **B** Transwell assays with and without Matrigel were performed to study cell migration or invasion. Scale bars: 100 μm. **C** EdU assays were performed to examine the proliferation of HTR-8/SVneo cells treated with recombinant BMP7 (50 ng/mL). Scale bars: 100 μm. **D** Flow cytometry assays were performed to examine the apoptosis rates of HTR-8/SVneo cells treated with recombinant BMP7 (50 ng/mL). **E**, **F** RNA-seq analysis of HTR-8/SVneo cells treated with or without recombinant BMP7 for 12 and 24 h. Principal component analysis (PCA, **E**) showing the separation between different groups. Venn diagram (**F**, left) and heatmap (**F**, right) showing an overlap of 691 differentially expressed genes (DEGs, adjusted *P* value < 0.05) between 12 h and 24 h BMP7 treatment, containing 158 genes with opposite changing trends and 533 genes with the same changing trends. **G** Heatmap depicting the top 50 common DEGs after both 12 h and 24 h of recombinant BMP7 treatment (adjusted *P* value < 0.05 and fold change contrast >1.2). **H**, **I** Kyoto Encyclopedia of Genes and Genomes (KEGG, **H**) pathway and Gene Ontology (GO, **I**) enrichment analysis of the transcriptome profiles of HTR-8/SVneo cells after BMP7 treatment. Data are presented as mean ± SD, and were analyzed by ANOVA (**A**) and unpaired two-tailed Student’s *t* tests (**B**–**D**). These data represent three independent experiments, each carried out in triplicate. *P* < 0.05 was considered statistically significant. ns not significant.

To identify the role of BMP7 in regulating human trophoblast function, HTR-8/SVneo cells were treated with recombinant BMP7 and subjected to wound healing and transwell assays. BMP7 promoted migration in a dose-dependent manner, peaking at 50 ng/mL (Fig. 2A), and consistently enhanced both migration and invasion (Fig. 2B). Importantly, BMP7 (50 ng/mL) did not affect cell proliferation or apoptosis, indicating that these effects are independent of altered cell viability (Fig. 2C, D).

To elucidate the molecular mechanisms underlying BMP7-promoted trophoblast migration and invasion, we performed bulk RNA sequencing (RNA-seq) on HTR-8/SVneo cells treated with or without BMP7 for 12 and 24 h. Principal component analysis (PCA) demonstrated clear segregation between BMP7-treated and control groups (Fig. 2E). Differential expression analysis identified 691 overlapping DEGs at both time points (Fig. 2F), including several notable trophoblast function- and PE-associated genes such as *ID1*, *ID2*, *SOX4*, *IGFBP3*, *FN1*, and *TGM2* [18, 30–33]. Among these, *TGM2*, which encodes tissue transglutaminase, was one of the most significantly downregulated genes following BMP7 treatment (Fig. 2G). Kyoto Encyclopedia of Genes and Genomes (KEGG) pathway analysis revealed that BMP7-treated cells exhibit enrichment in pathways associated with PE, including PI3K-Akt, MAPK, and TGF-β signaling pathways (Fig. 2H) [34–36]. Gene Ontology (GO) analysis further highlighted significant changes in genes related to extracellular matrix organization, regulation of angiogenesis, cell-substrate adhesion, epithelial cell migration, and epithelial-to-mesenchymal transition (Fig. 2I). Together, these results suggest that BMP7 plays a crucial role in promoting trophoblast migration and invasion, likely through modulating key signaling pathways and cellular processes relevant to PE development.

### TGM2 is a downstream target of BMP7 and is upregulated in PE placentas

**Fig. 3:**
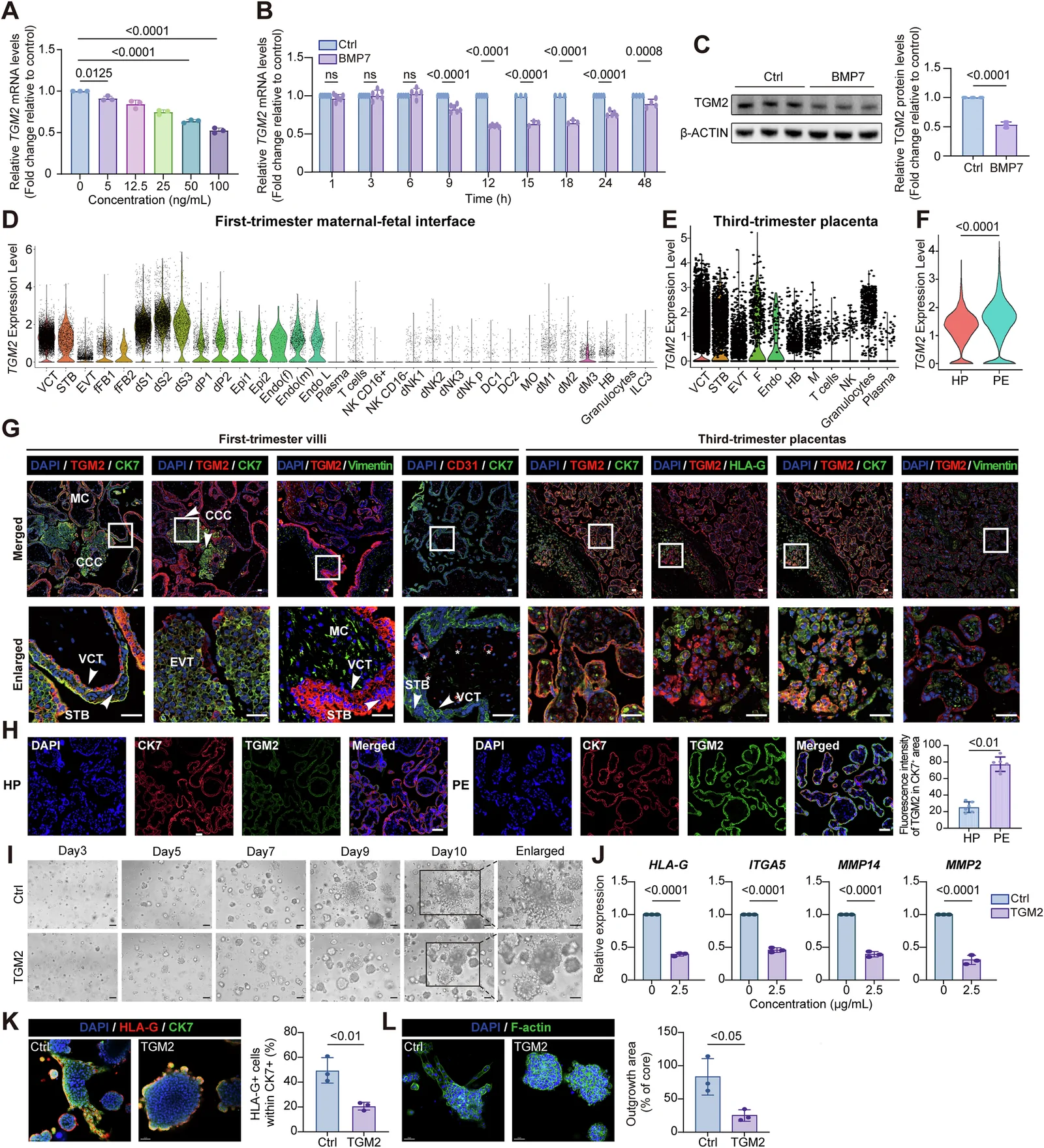
TGM2 expression is upregulated in preeclamptic placentas, and recombinant BMP7 treatment decreases the expression of TGM2. **A** HTR-8/SVneo cells were treated with different concentrations of recombinant BMP7, and *TGM2* mRNA levels were measured by RT-qPCR. **B** RT-qPCR analysis was performed to measure mRNA levels of *TGM2* in HTR-8/SVneo cells treated with or without 50 ng/mL recombinant BMP7 for different durations. **C** Western blot was performed to analyze TGM2 protein levels in HTR-8/SVneo cells treated with or without 50 ng/mL recombinant BMP7 for 48 h. The left panel shows a representative picture, and the right panel shows the summarized quantitative result. **D**, **E** Violin plots showing cell-type-specific expression of TGM2 in scRNA-seq datasets from the first-trimester maternal-fetal interface (**D**) and third-trimester placentas (**E**). Strong expression is observed in VCTs. **F** Violin plot comparing TGM2 expression in VCTs between 5 preeclamptic (PE) and 5 healthy (HP) third-trimester placentas. **G** Immunofluorescence localization of TGM2 in first-trimester villi and third-trimester human placental tissues. Asterisks indicate blood vessels. Scale bars: 50 μm. **H** Immunofluorescence staining of CK7 and TGM2 in third-trimester human placental villous tissues from 6 PE donors and 6 HP donors. Representative images are shown, and the fluorescence intensity of TGM2 was quantified in the CK7-positive trophoblast compartment. Scale bars: 50 μm. **I** Representative bright-field images of trophoblast organoids cultured in EVT differentiation medium with or without recombinant TGM2 (μg/mL) for the indicated days (Day 3–10). Scale bars: 100 μm. **J** RT-qPCR analysis of EVT/invasion-associated markers (HLA-G, ITGA5, MMP14, MMP2) in control versus TGM2-treated organoids. **K** Immunofluorescence staining of HLA-G and CK7 in control and TGM2-treated trophoblast organoids. Representative images are presented in the left panel, and the quantitative results showing the proportion of HLA-G^+^ cells among CK7^+^ cells are presented in the right panel. Scale bars: 50 μm. **L** Confocal staining for F-actin in control and TGM2-treated trophoblast organoids. Representative images are presented in the left panel, and the quantitative analysis of outgrowth area relative to organoid core size is presented in the right panel. Scale bars: 50 μm. Data represented as mean ± SD. *P* value was calculated by ANOVA (**A**, **B**) and unpaired two-tailed Student’s *t* tests (**C**, **F**, **H**, **J**–**L**). *P* < 0.05 was considered statistically significant. ns not significant. These data represent three independent experiments, each carried out in triplicate.

Elevated TGM2 expression in PE placentas have been reported previously, although its role in regulating trophoblast behavior is still unknown [18]. To validate *TGM2* as a downstream target of BMP7, as identified in our RNA-seq analysis, we treated HTR-8/SVneo cells with BMP7 at different dosages and time points (Fig. 3A, B). Quantitative real-time reverse-transcription PCR (RT‒qPCR) analysis showed a significant reduction in *TGM2* mRNA expression after BMP7 treatment, with the effect becoming more pronounced after 12–18 h of treatment and with increasing BMP7 concentrations (Fig. 3A, B). Consistent with the transcriptional changes, western blot analysis demonstrated a marked decrease in TGM2 protein levels after 48 h of BMP7 treatment in HTR-8/SVneo cells (Fig. 3C).

ScRNA-seq analysis revealed cell-type-specific expression of *TGM2*, with strong enrichment in VCTs in both the first-trimester maternal-fetal interface and third-trimester placentas (Fig. 3D, E) [24–26]. Consistent with previous reports of elevated TGM2 in PE placentas, scRNA-seq data further showed significantly higher *TGM2* expression in VCTs from PE patients compared to HP controls (Fig. 3F). Immunostaining confirmed abundant TGM2 localization in trophoblasts within first-trimester villi and third-trimester placentas (Fig. 3G). Co-staining for CK7 and TGM2 demonstrated significantly increased TGM2 expression in trophoblasts from PE placentas compared to HP placentas (Fig. 3H). This increase was further supported by RT-qPCR and western blot analyses of placental villous tissue, which demonstrated higher TGM2 expression in PE placental villi than in control placental villi (Fig. S1C, D).

Collectively, these findings identify TGM2 as a downstream effector of BMP7 signaling, with aberrant upregulation of trophoblastic TGM2 likely mediating the effects of BMP7 deficiency, thereby impairing trophoblast function and contributing to PE pathogenesis.

### TGM2 restricts acquisition of an invasive extravillous trophoblast phenotype in human trophoblast organoids

Acquisition of an invasive phenotype marks the differentiation of CTBs into EVTs, and its impairment results in shallow trophoblast invasion, a hallmark of PE pathogenesis [9]. To examine the role of TGM2 in trophoblast invasiveness and early placentation ex vivo, we established human trophoblast organoids (TOs) under an EVT-induction paradigm and quantified invasive differentiation with or without recombinant TGM2 protein treatment (Fig. S1E, F). Because TGF-β inhibitors are required for organoid culture, direct assessment of BMP7 function was not feasible in this system.

Primary trophoblasts isolated from first-trimester villi of healthy pregnancies were cultured in trophoblast organoid medium (TOM) for 3–4 days, then transferred to EVT differentiation medium (EVT medium) with or without recombinant TGM2 (2.5 μg/mL) for the indicated period (Fig. 3I). RT–qPCR revealed reduced expression of EVT markers, including *HLA-G*, *ITGA5*, *MMP14*, and *MMP2*, in TGM2-treated organoids (Fig. 3J). Immunofluorescence corroborated impaired invasive differentiation, with markedly decreased HLA-G expression, a hallmark of EVTs (Fig. 3K). F-actin/DAPI staining consistently showed a significant reduction in organoid outgrowth area (expressed as % of core) in the TGM2 group, indicating diminished emergence of invasive trophoblasts from the organoid body (Fig. 3L).

Collectively, these data indicate that elevated TGM2 restrains EVT differentiation and outgrowth in trophoblast organoids, providing a potential mechanistic link between increased trophoblastic TGM2 in preeclamptic placentas and defective trophoblast invasion during PE pathogenesis.

### BMP7 downregulates TGM2 expression through an ALK2/ALK3-SMAD1/5/9-SMAD4-dependent pathway

**Fig. 4:**
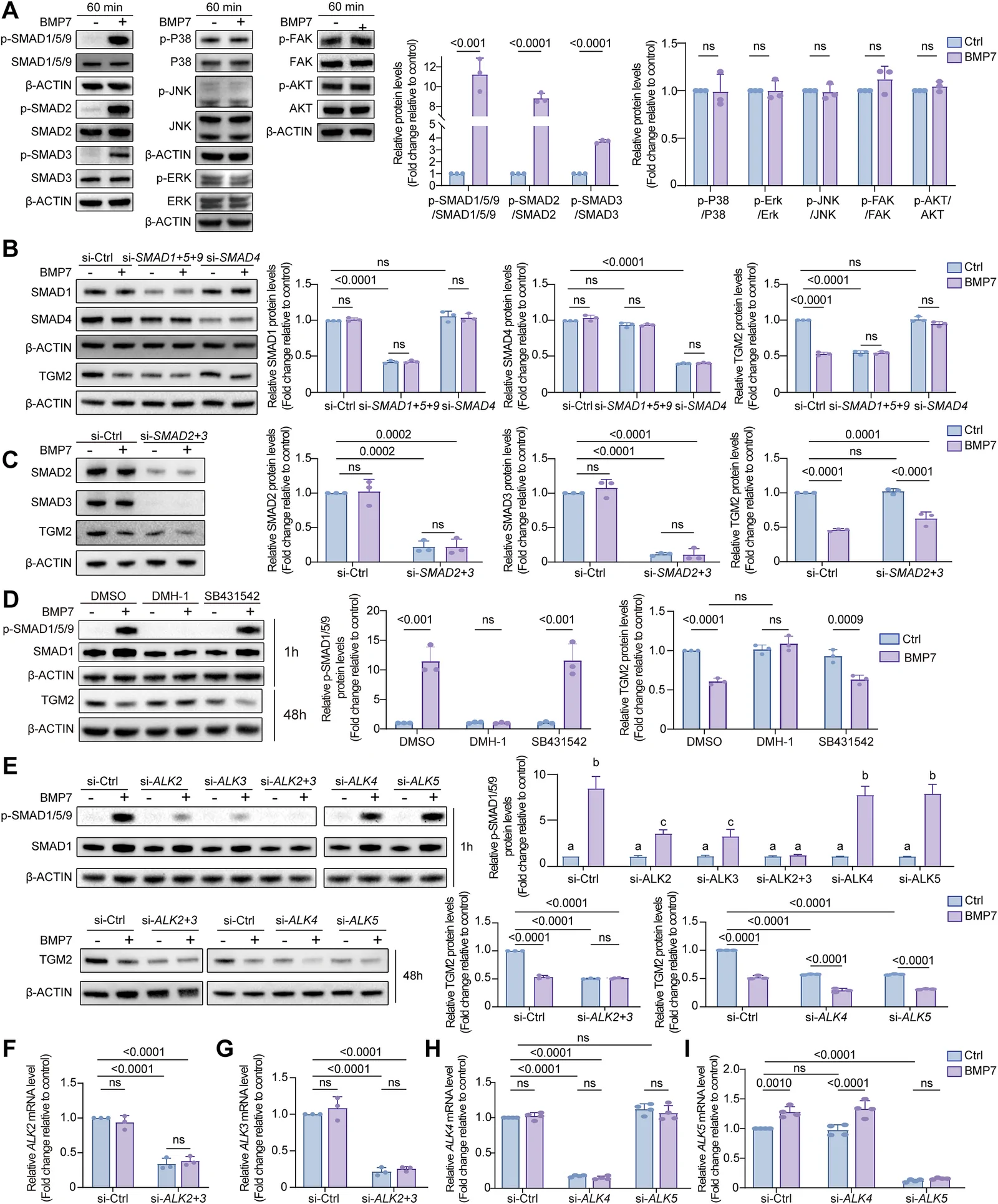
BMP7 downregulates TGM2 through ALK2/3-SMAD1/5/9-SMAD4 in HTR-8 cells. **A** HTR-8/SVneo cells were treated with or without 50 ng/mL recombinant BMP7 for 60 min. The protein levels of the targets were assayed by western blot. **B** HTR-8/SVneo cells were transfected for 48 h with 20 nM control non-targeting siRNA, 20 nM siRNA targeting *SMAD1/5/9* (*SMAD1* + *SMAD5* + *SMAD9*), or 20 nM siRNA targeting *SMAD4* before treatment with or without 50 ng/mL recombinant BMP7 for 48 h. The protein levels of the targets were assayed by western blot. **C** HTR-8/SVneo cells transfected for 48 h with 20 nM control non-targeting siRNA or 20 nM siRNA targeting *SMAD2/3* (*SMAD2* + *SMAD3*) before treatment with or without 50 ng/mL recombinant BMP7 for 48 h. The protein levels of SMAD2, SMAD3, and TGM2 were analyzed by western blot. **D** HTR-8/SVneo cells pretreated with vehicle (DMSO), the ALK2 and ALK3 inhibitor DMH-1 (1 μM), or the ALK4, ALK5, and ALK7 inhibitor SB431542 (10 μM) for 60 min, followed by treatment with or without BMP7 (50 ng/mL) for 1 h or 48 h. Protein levels were assayed by western blot. **E** HTR-8/SVneo cells transfected with 20 nM non-targeting control siRNA or siRNAs targeting *ALK2* and/or *ALK3* or *ALK4* or *ALK5* for 48 h before treatment with or without recombinant BMP7 (50 ng/mL) for 1 h (upper panel) or 48 h (lower panel). The summarized quantitative results for p-SMAD1/5/9 and TGM2 protein levels are shown in the panel. **F**–**I** HTR-8/SVneo cells were transfected with 20 nM non-targeting control siRNA or siRNAs targeting *ALK2* and/or *ALK3* or *ALK4* and *ALK5* for 48 h before treatment with or without recombinant BMP7 (50 ng/mL). RT-qPCR was used to examine the *ALK2*, *ALK3*, *ALK4*, and *ALK5* mRNA levels. Data are represented as mean ± SD. *P* value was calculated by unpaired two-tailed Student’s *t* tests (**A**) and ANOVA (**B**–**I**). *P* < 0.05 was considered statistically significant. ns, not significant. Columns without letters in common are significantly different (**E**). ns not significant. These data represent three independent experiments, each carried out in triplicate.

To elucidate the signaling pathways underlying BMP7-mediated regulation of TGM2 expression, we assessed pathway activation in HTR-8/SVneo cells following a 60-min treatment with BMP7 (50 ng/mL). Western blot analysis revealed that BMP7 significantly increased the phosphorylation of SMAD1/5/9 and SMAD2/3. In contrast, BMP7 had no effect on the phosphorylation of key non-canonical signaling proteins, including P38, JNK, ERK, FAK, or AKT (Fig. 4A).

To determine the mediating SMAD pathway, HTR-8/SVneo cells were transfected with siRNAs targeting *SMAD1/5/9*, *SMAD2/3*, or *SMAD4*. Following validated knockdown, silencing SMAD1/5/9 or SMAD4, but not SMAD2/3, significantly attenuated BMP7-induced TGM2 suppression (Fig. 4B, C), indicating that BMP7 downregulates TGM2 through the canonical SMAD1/5/9-SMAD4 pathway.

To identify the type I TGF-β receptors responsible for BMP7-induced SMAD1/5/9 phosphorylation and TGM2 downregulation [9], HTR-8/SVneo cells were treated with either DMH-1 (an inhibitor of ALK2, ALK3, and ALK6) or SB431542 (an inhibitor of ALK4, ALK5, and ALK7) prior to BMP7 treatment. DMH-1 completely abolished BMP7-induced SMAD1/5/9 phosphorylation and TGM2 downregulation, whereas SB431542 had no effect (Fig. 4D). Receptor specificity analysis revealed that individual knockdown of ALK2 or ALK3 attenuated BMP7-induced SMAD1/5/9 phosphorylation, whereas combined ALK2/3 knockdown completely abolished SMAD1/5/9 activation and TGM2 downregulation (Fig. 4E–I).

These findings were further corroborated in primary human trophoblast cells isolated from first-trimester villi (Fig. S2A–D), whose purity was confirmed by CK7 immunostaining (Fig. S2E). Notably, knockdown of ALK receptors or SMAD proteins alone altered basal TGM2 expression, suggesting that its steady-state levels involve a complex multifactorial network. Despite these baseline effects, experiments in both HTR8/SVneo cells and primary human trophoblast cells demonstrated that ALK2 and ALK3 serve as the principal type I receptors mediating BMP7-induced SMAD1/5/9 phosphorylation and subsequent TGM2 downregulation in trophoblasts.

To further characterize the expression pattern of ALK2 and ALK3 at the maternal-fetal interface, scRNA-seq analysis was performed, revealing pronounced expression of ALK2 and ALK3 in VCTs at the first-trimester maternal-fetal interface (Fig. S3A, B). Exploratory comparison of ALK2^+^ALK3^+^ and ALK2^-^ALK3^-^ VCTs showed higher expression of canonical BMP-responsive genes, including *ID1* and *ID2*, together with lower *TGM2* expression in the ALK2^+^ALK3^+^ subset (Fig. S3C). In addition, several trophoblast function-related genes, including *SVEP1*, *ADAMTS6*, and *HBEGF*, were enriched in the ALK2^+^ALK3^+^ subset (Fig. S3D). These findings provide further indirect evidence that ALK2/3 receptors contributes to the regulation of trophoblast phenotype [37–42].

### TGM2 mediates BMP7-regulated trophoblast migration and invasion

**Fig. 5:**
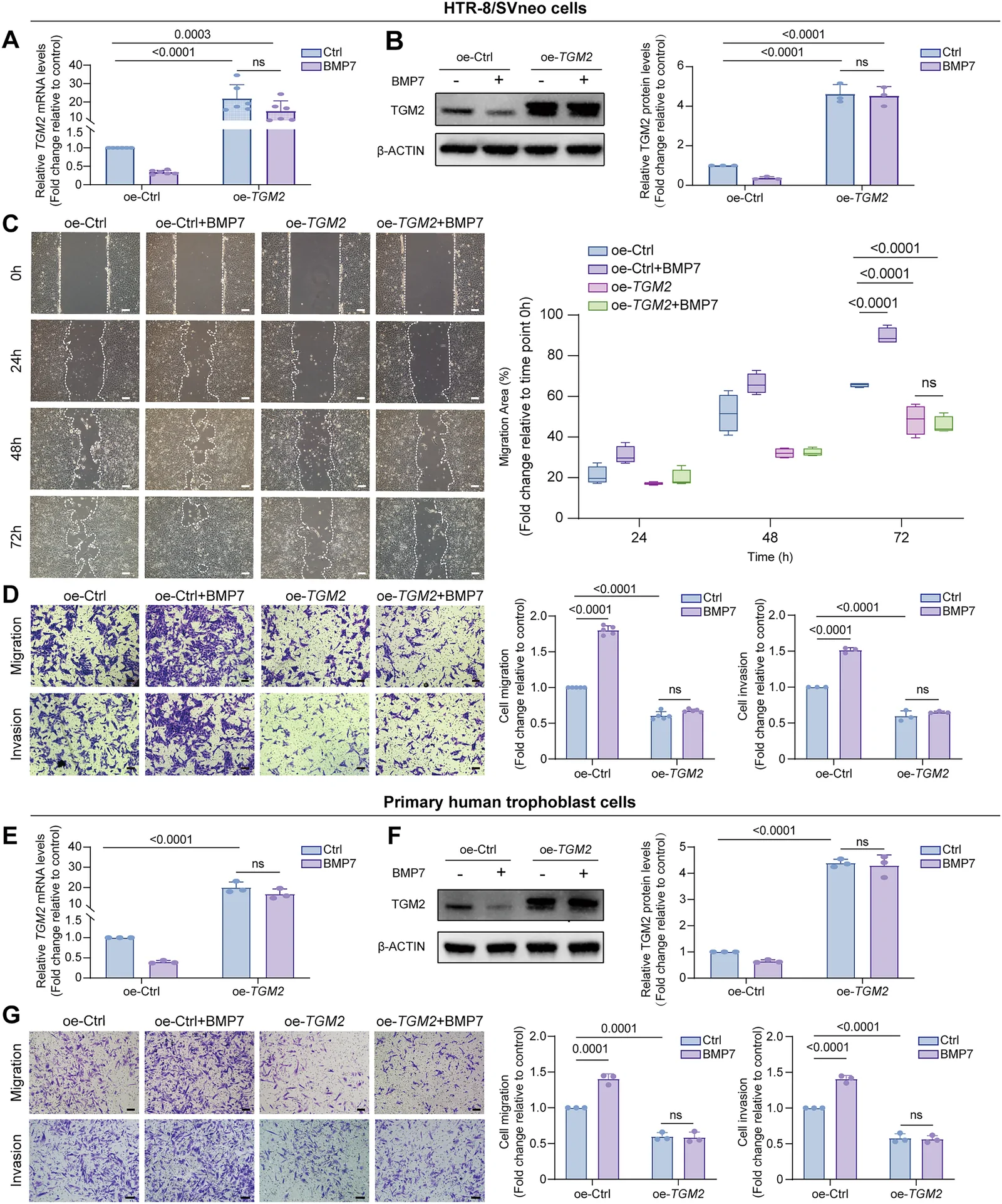
TGM2 mediates basal and BMP7-promoted HTR-8/SVneo cells and primary human trophoblast cells migration and invasion. **A**, **B** HTR-8/SVneo cells were transfected for 48 h with a vector plasmid (oe-Ctrl) or TGM2-overexpressing plasmid (oe-TGM2) before treatment with or without 50 ng/mL recombinant BMP7. *TGM2* mRNA levels (**A**) were examined by qPCR after BMP7 treatment for 12 h in HTR-8/SVneo cells. TGM2 protein levels (**B**) after transfection for 48 h before treatment with or without recombinant BMP7 were assessed via western blot in HTR-8/SVneo cells. **C** Wound-healing assays were used to measure cell mobility. Scale bars: 100 μm. **D** Transwell assays with or without Matrigel were performed to measure cell migration and invasion. Representative images are presented in the left panel, and the summarized quantitative results are presented in the right panel. Scale bars: 100 μm. **E** *TGM2* mRNA levels were examined by qPCR after transfection with a vector plasmid (oe-Ctrl) or TGM2-overexpressing plasmid (oe-TGM2) before treatment with or without 50 ng/mL recombinant BMP7 for 48 h in primary human trophoblast cells. **F** TGM2 protein levels in primary human trophoblast cells after transfection with a vector plasmid (oe-Ctrl) or a TGM2-overexpressing plasmid (oe-TGM2) before treatment with or without 50 ng/mL recombinant BMP7 for 48 h. **G** Transwell assays with or without Matrigel were performed to measure cell migration and invasion in primary human trophoblast cells. Scale bars: 100 μm. Data are represented as mean ± SD. *P* value was calculated by ANOVA. *P* < 0.05 was considered statistically significant. ns, not significant. These data represent three independent experiments, each carried out in triplicate.

To determine whether TGM2 downregulation is required for the pro-migratory and pro-invasive effects of BMP7, we overexpressed TGM2 in HTR-8/SVneo cells via plasmid transfection followed by BMP7 treatment. RT-qPCR and Western blot analysis confirmed successful overexpression of TGM2 at both the mRNA and protein levels (Fig. 5A, B). Functional assays, including wound healing and transwell assays, demonstrated that TGM2 overexpression significantly inhibited both basal and BMP7-enhanced HTR-8/SVneo cell migration and invasion (Fig. 5C, D).

To confirm these findings in a physiologically relevant context, we utilized primary human trophoblast cells isolated from first-trimester chorionic villi. TGM2 overexpression was verified by RT-qPCR and Western blot analysis (Fig. 5E, F). Consistent with the results in HTR-8/SVneo cells, recombinant BMP7 enhanced migration and invasion of primary human trophoblast cells, while TGM2 overexpression attenuated BMP7-induced migratory and invasive abilities (Fig. 5G). EdU incorporation and flow cytometry assays further demonstrated that neither BMP7 treatment (50 ng/mL) nor TGM2 overexpression affected cell proliferation, underscoring the specific role of BMP7-TGM2 signaling in regulating trophoblast migration and invasion (Fig. S4A–C). Together, these results suggest TGM2 as a downstream effector of BMP7 that mediates its control of trophoblast motility.

To further validate the mediating role of TGM2 in BMP7-regulated trophoblast function, we performed loss-of-function experiments in primary human trophoblast cells using specific siRNAs to knock down BMP7 or TGM2, respectively. BMP7 knockdown increased endogenous TGM2 expression and impaired trophoblast migration and invasion (Fig. S5A–F). Conversely, TGM2 knockdown enhanced trophoblast migration and invasion and largely attenuated the additional pro-migratory and pro-invasive effect of BMP7 (Fig. S5G–L). Together, these data further support TGM2 as a functionally relevant downstream effector of BMP7 in trophoblasts.

### Recombinant BMP7 protein administration alleviates PE-related symptoms in an Ad sFlt-1-induced PE mouse model

**Fig. 6:**
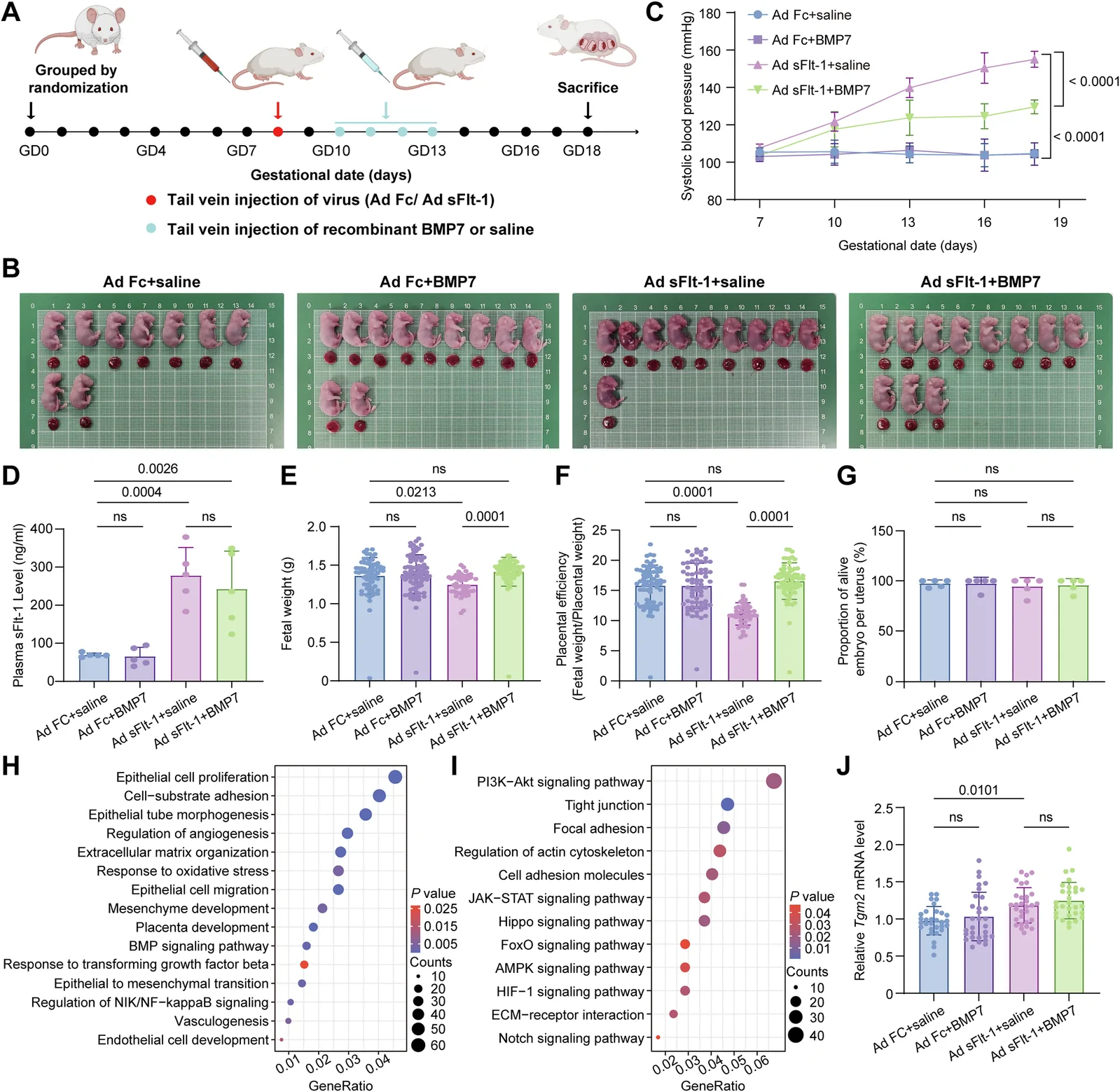
Supplementation with recombinant BMP7 protein improved pregnancy outcomes in Ad sFlt-1-induced PE mice. **A** Schematic diagram illustrating the animal experimental protocol. Adenoviruses expressing sFlt-1 or control Fc were injected into the tail vein of CD-1 mice at GD8. The pregnant mice were then subjected to tail vein recombinant BMP7 protein (10 μg/kg/d) or saline injection daily from GD10 to GD13. Blood pressure was measured and recorded every 2‒3 days from GD7 to GD18. Mice were harvested on GD18. **B** Images of mouse fetuses and placentas at GD18 in each group. **C** Systolic blood pressure (SBP) of mice from each group. **D** Serum sFlt-1 levels at GD18. **E** The fetal weight of each group at GD18. **F** Ratio of fetal/placental weights from each group. **G** Proportion of living embryos in each group. **H**, **I** GO (**H**) and KEGG (**I**) analyses of the DEGs (adjusted *P* value < 0.05 and fold change contrast >1.2). **J** Relative *Tgm2* mRNA levels in placentas from different groups. *n* = 6 placentas per mouse. *N* = 5. *N* indicates the number of individual animals in each group. ns not significant. Data are represented as mean ± SD. *P* value was calculated by ANOVA. *P* < 0.05 was considered statistically significant. ns not significant.

Based on the observed trophoblastic BMP7 deficiency in preeclamptic placentas and the demonstrated functional role of BMP7 in trophoblasts, we next explored the therapeutic potential of BMP7 in PE. To this end, pregnancy outcomes were evaluated following recombinant BMP7 protein supplementation in an Ad sFlt-1-induced PE mouse model [21]. Mice receiving the same dose of adenovirus carrying the murine immunoglobulin G2α Fc fragment served as controls (Ad Fc group). Pregnant mice were randomly assigned to four groups on gestational day (GD) 0, including Ad Fc+saline group, Ad Fc+BMP7 group, Ad sFlt-1+saline group, and Ad sFlt-1 + BMP7 group, followed by treatment with adenovirus on GD8. PE-related pathological features, including blood pressure, fetal weight, placental weight, serum sFlt-1 levels, and histological changes in placenta and kidney were assessed on GD18 (Fig. 6A). Initial body weights at GD0 and GD8 were comparable across all groups (Fig. S6A).

Intravenous injection of adenovirus carrying sFlt-1 successfully established the PE-like mouse model (Fig. 6B). Mice in the Ad sFlt-1+saline group exhibited significantly higher systolic blood pressure (SBP) (Fig. 6C) and serum sFlt-1 (Fig. 6D) levels, reduced fetal weights (Fig. 6E), and decreased placental efficiency (fetal/placental weight ratio) compared to the Ad Fc+saline control mice (Fig. 6F), although the proportion of live embryos remained similar between groups (Fig. 6G). RNA-seq analysis revealed global transcriptome changes in the placentas of the Ad sFlt-1+saline group compared to the Ad Fc+saline group, with clear separation in PCA plot (Fig. S6B). KEGG pathway analysis indicated enrichment in PI3K-Akt signaling, focal adhesion, JAK-STAT signaling, and HIF-1 signaling, while GO term revealed alterations in epithelial cell proliferation, migration, epithelial to mesenchymal transition, vasculogenesis, oxidative stress response, and BMP signaling (Fig. 6H, I). Notably, *Tgm2* was among the top upregulated DEGs in the PE model (Fig. S6C), mirroring the trend observed in human preeclamptic placentas. Consistently, RT-qPCR analysis confirmed significantly elevated placental *Tgm2* mRNA levels in the Ad sFlt-1+saline group compared to the Ad Fc+saline group (Fig. 6J).

**Fig. 7:**
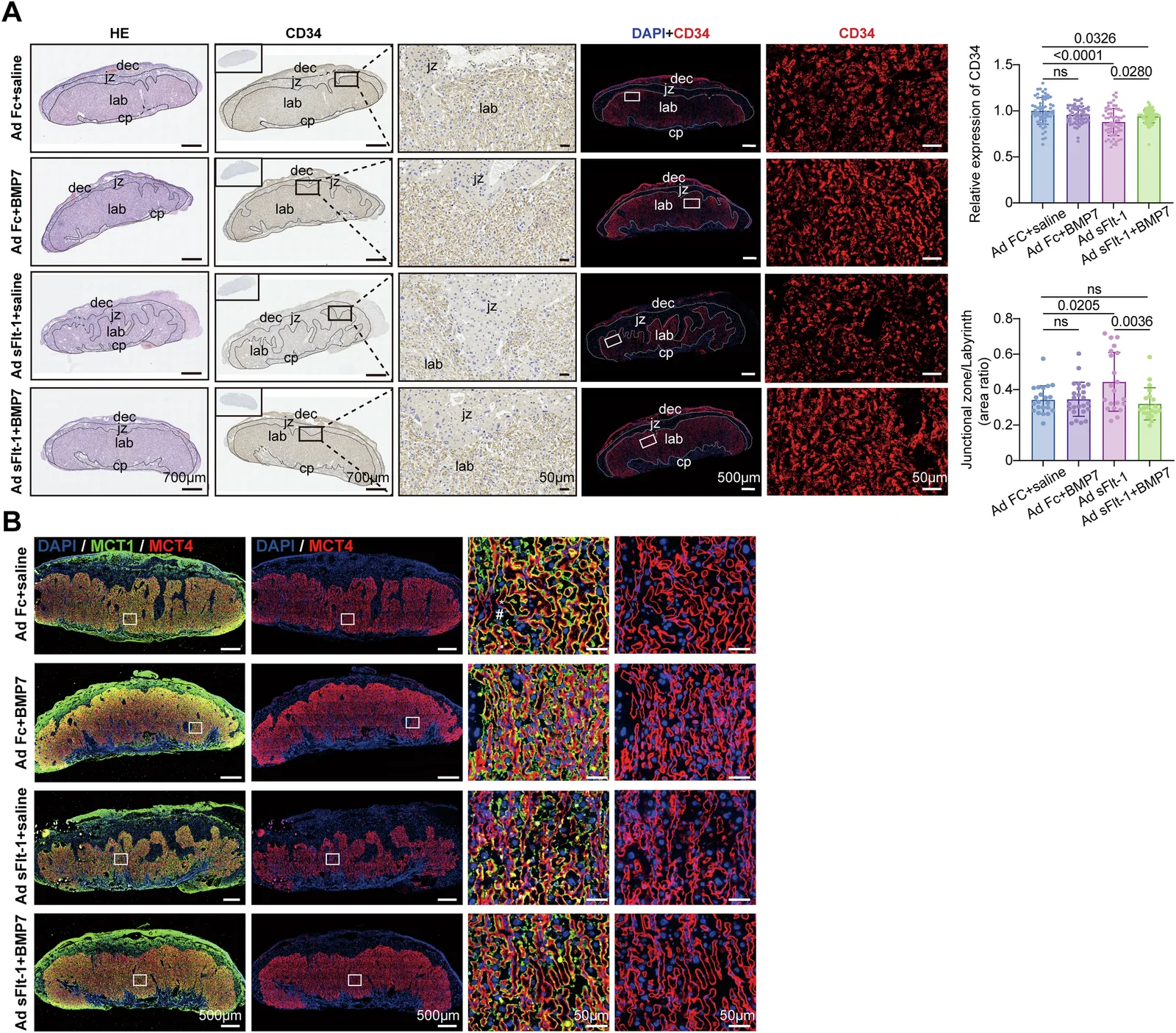
Supplementation with recombinant BMP7 improved placental morphology in PE mice. **A** Structural and quantitative analyses of mouse placentas at GD18. Left: H&E staining, IHC, and IF staining for CD34 in representative placental sections (dec, decidua; jz, junctional zone; lab, labyrinth; cp, chorionic plate). Right, upper: signal intensity of CD34 IHC quantified with ImageScope (*n* = 12 regions of the placental labyrinth per mouse). Right, lower: ratios of junctional zone and labyrinth areas quantified and summarized (4–5 placentas per animal in each group). **B** IF staining of MCT1 and MCT4 in mouse placentas at GD18. MCT1 is specifically expressed in the SynT-I layer that faces maternal blood sinuses, whereas MCT4 specifically stains the SynT-II layer that encloses fetal blood vessels. Asterisks and numbers indicate fetal blood vessels and maternal blood sinuses, respectively. Each group includes five individual animals. Data are represented as mean ± SD. *P* value was calculated by ANOVA. *P* < 0.05 was considered statistically significant. ns not significant.

Histological analysis revealed structural alterations in placentas. Immunostaining displayed changes in overall placental architecture, while CD34 staining, used to assess fetal and maternal vasculature, demonstrated impaired placental development in the Ad sFlt-1+saline group. The junctional zone and labyrinth ratios were altered, with reduced and sparser CD34 staining observed in the Ad sFlt-1+saline group compared to controls (Fig. 7A). Immunofluorescence staining of MCT1 and MCT4—markers of the syncytin I (SynT-I) and syncytin II (SynT-II) layers of the labyrinth, respectively—revealed a marked reduction of the MCT4^+^ SynT-II layer (Fig. 7B), indicative of impaired development of the fetal-maternal exchange interface [43]. Additionally, renal damage, another hallmark of PE, was evidenced by HE, PAS and CD34 staining, which demonstrated glomerular endothelial injury in the Ad sFlt-1+saline group (Fig. S6D, E) [44, 45].

Importantly, BMP7 supplementation significantly alleviated PE-related phenotypes, including increased SBP, decreased fetal weight, as well as placental and renal damage in the sFlt-1 + BMP7 group, without affecting serum sFlt-1 levels (Figs. 6B–G, 7 and S6D, E), which supports the therapeutic potential of BMP7 in PE. However, this improvement was not accompanied by a reduction in total placental *Tgm2* mRNA, suggesting that the protective effect of BMP7 in vivo through mechanisms beyond total placental Tgm2 expression alone.

To more directly assess the pathogenic contribution of TGM2 in vivo, as implicated by our in vitro findings, we used a pharmacological inhibition approach. Cystamine diHCl, a transglutaminase inhibitor commonly used to inhibit TGM2 activity, was administered to sFlt-1-induced PE mice (Fig. S7A). Notably, cystamine diHCl treatment markedly alleviated PE-like manifestations in this model (Fig. S7B). Compared with vehicle-treated disease controls, cystamine diHCl-treated sFlt-1 mice exhibited significantly reduced systolic blood pressure (Fig. S7C), along with improved fetal weight and placental efficiency (Fig. S7D–F). These findings support a pathogenic role for hyperactive TGM2 in the PE-like phenotype of this model.

## Discussion

**Fig. 8:**
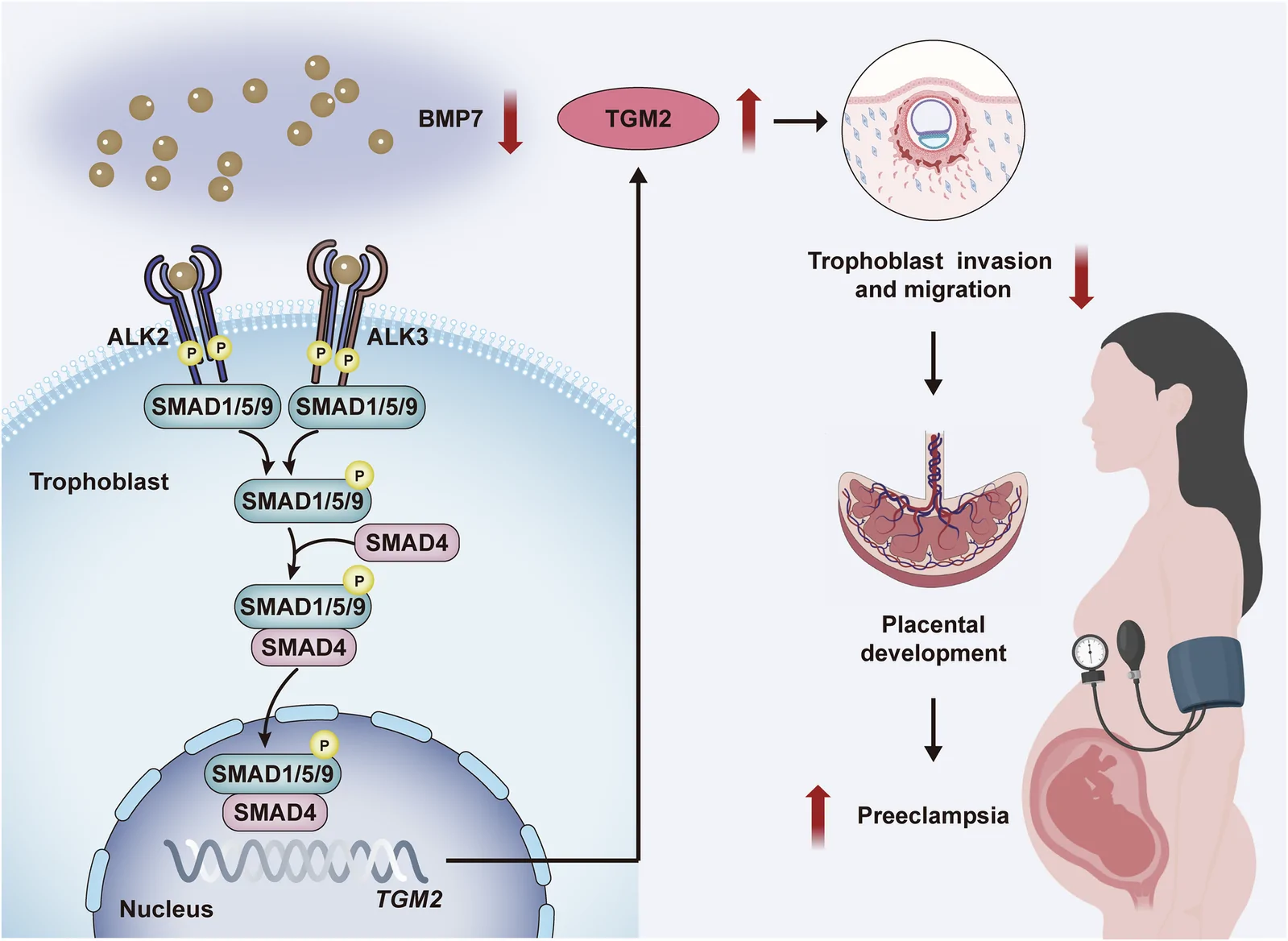
The schematic diagram illustrates the role of the dysregulated BMP7-TGM2 axis in PE pathogenesis. In normal pregnancy, BMP7 suppresses TGM2 expression through the ALK2/ALK3-SMAD1/5/9-SMAD4 signaling pathway, thereby promoting trophoblast migration and invasion. In PE placentas, reduced BMP7 leads to TGM2 upregulation, which limits trophoblast invasion and impairs placental remodeling, contributing to disease pathogenesis.

In this study, we identified significantly reduced BMP7 and elevated TGM2 expression in trophoblasts from preeclamptic placentas, implicating their dysregulation in PE pathogenesis. To our knowledge, this study provides the first evidence that BMP7 regulates trophoblast migration and invasion through TGM2 as a functionally relevant downstream effector. Mechanistically, BMP7 suppresses TGM2 expression via ALK2/ALK3 receptors through activation of the SMAD1/5/9-SMAD4 signaling pathway (Fig. 8). Using a human trophoblast organoid model, we further demonstrated an anti-invasive role for TGM2 during early placental differentiation. Consistent with the human data, placental *Tgm2* expression was elevated in an Ad sFlt-1-induced PE mouse model, while recombinant BMP7 supplementation significantly alleviated PE-like symptoms, improving placental efficiency. Together, these findings identify the BMP7-TGM2 signaling axis as an important regulator of trophoblast behavior and highlight BMP7 as a potential therapeutic candidate for PE.

Previous studies have shown that BMP7 promotes migration and invasion in several cancer types [46, 47]. In contrast to its pathological role in tumor metastasis, our findings reveal a physiological role for BMP7 in promoting trophoblast migration and invasion during placental development. VCTs were identified as the predominant BMP7-expressing population in both first-trimester villi and third-trimester placentas. In maternal serum, BMP7 levels were undetectable regardless of trimester or PE status, suggesting its activity is largely confined to the placental microenvironment and mediated through local autocrine/paracrine signaling.

TGM2 is abundantly expressed in the placenta, with reduced levels in invasive trophoblasts compared to villous populations [48]. Previous research has reported elevated TGM2 expression in preeclamptic placentas, implicating its role in PE pathogenesis through isopeptide modification and AT1 receptor stabilization [18]. Consistent with these reports, we observed not only an overall increase in total placental TGM2 levels but also a specific upregulation of TGM2 in villous cytotrophoblasts from preeclamptic placentas. Along with the increased placental *Tgm2* mRNA in the Ad sFlt-1-induced PE mouse model, these findings support a conserved contribution of TGM2 upregulation to PE pathophysiology.

Consistent with in vivo findings, recombinant TGM2 treatment in the human trophoblast organoid model suppressed EVT differentiation and outgrowth, supporting an inhibitory role for elevated TGM2 in trophoblast invasion-related behavior. Future studies are warranted to determine whether trophoblastic TGM2 acts predominantly through intracellular mechanisms or through cell-surface and extracellular matrix-associated functions [17]. This inhibitory role of TGM2 aligns closely with that of TGF-β1, a well-established suppressor of trophoblast invasion known to induce TGM2 expression [19, 49]. Elevated TGF-β1 levels in both the serum and placental tissue of PE patients [50–52], along with evidence of TGF-β1-driven fibrosis in PE placentas [53], further underscore its pathological relevance. In contrast, BMP7 often antagonizes TGF-β1 signaling [54], with dysregulation of this balance implicated in diseases such as hepatocellular carcinoma [55]. Consistent with the broader diversity of TGF-β superfamily functions, whereas TGF-βs and Nodal inhibit trophoblast invasion, activins and BMPs promote it, suggesting that precise equilibrium among these pathways is essential for normal placentation [9]. Because TGM2 is transcriptionally regulated by both TGF-β1 and BMP7, it may serve as a downstream integrator that balances pro- and anti-invasive cues to determine the appropriate extent of trophoblast invasion, warranting further investigation.

Although no animal model fully replicates the complexity of human PE, our use of the Ad sFlt-1-induced model recapitulates hallmark PE features [21], including hypertension and placental abnormalities. In this PE-like mice model, recombinant BMP7 administration significantly mitigated PE-like phenotypes, yet bulk placental transcriptomic analysis did not show a marked reduction in *Tgm2* mRNA after BMP7 treatment. Given the marked cellular heterogeneity of the placenta, bulk tissue analysis may mask trophoblast-specific regulation. Moreover, our in vivo rescue experiments with cystamine diHCl indicate that TGM2 hyperactivity makes a pathogenic contribution to this model. These findings suggest that BMP7 may inhibit TGM2 in a localized manner. Given the pleiotropic nature of BMP7, its therapeutic effects in vivo are also likely mediated through additional protective pathways beyond TGM2 suppression alone. Our previous work demonstrated that another FDA-approved BMP, BMP2, similarly alleviated PE symptoms in rats, enhancing trophoblast invasion by inducing BMP6 via SMAD2/3 and activin A via SMAD1/5/8 [23, 56]. Here, we show that BMP7 activates both SMAD1/5/9 and SMAD2/3 pathways, yet only SMAD1/5/9 mediates TGM2 suppression. These findings raise the possibility that BMP2 and BMP7 may act synergistically to restore placental function by converging on complementary trophoblast regulatory networks. Future studies should explore whether combined or sequential administration of BMP2 and BMP7 enhances therapeutic efficacy. Moreover, advances in placenta-targeted drug delivery systems, including nanoparticle-based formulations and ligand-directed approaches, offer promising strategies to deliver BMP therapeutics with improved safety and efficacy [22, 57].

In conclusion, through integrated single-cell, in vitro, ex vivo, and in vivo analyses, we define a dysregulated BMP7–TGM2 signaling axis as an important driver of impaired trophoblast invasion in PE. Restoration of BMP7 signaling ameliorates PE-like pathological features in vivo, supporting a mechanistic and translational foundation for BMP7-based therapeutic development. More broadly, these findings provide further insight into the contribution of BMP signaling to placental homeostasis and highlight the therapeutic potential of targeting trophoblastic signaling pathways in PE.

## Supplementary information

Clean Supplementary Manuscript

Uncropped western blots

## Data Availability

All methods and data supporting the findings of this study are available within the manuscript and its supplementary information. All bioinformatic analyses were performed using publicly available software as described in “Methods”. The RNA-seq data were deposited in the NCBI SRA database (www.ncbi.nlm.nih.gov/subs/sra/) under the accession code PRJNA1175654 (cell line data) and PRJNA1175369 (mouse placentas data).

## References

[1] Chappell LC, Cluver CA, Kingdom J, Tong S. Pre-eclampsia. Lancet. 2021;398:341–54. 10.1016/S0140-6736(20)32335-7 34051884

[2] Say L, Chou D, Gemmill A, Tunçalp Ö, Moller A-B, Daniels J, et al. Global causes of maternal death: a WHO systematic analysis. Lancet Glob Health. 2014;2:e323–33. 10.1016/S2214-109X(14)70227-X 25103301

[3] ACOG. Gestational hypertension and preeclampsia: ACOG Practice Bulletin, Number 222. Obstet Gynecol. 2020;135:e237. 10.1097/AOG.0000000000003891 32443079

[4] Davies JE, Pollheimer J, Yong HEJ, Kokkinos MI, Kalionis B, Knöfler M, et al. Epithelial-mesenchymal transition during extravillous trophoblast differentiation. Cell Adhes Migr. 2016;10:310–21. 10.1080/19336918.2016.1170258 PMC4951171 27070187

[5] Arutyunyan A, Roberts K, Troulé K, Wong FCK, Sheridan MA, Kats I, et al. Spatial multiomics map of trophoblast development in early pregnancy. Nature. 2023;616:143–51. 10.1038/s41586-023-05869-0 PMC10076224 36991123

[6] Castel G, Meistermann D, Bretin B, Firmin J, Blin J, Loubersac S, et al. Induction of human trophoblast stem cells from somatic cells and pluripotent stem cells. Cell Rep. 2020;33:108419. 10.1016/j.celrep.2020.108419 33238118

[7] Rana S, Lemoine E, Granger JP, Karumanchi SA. Preeclampsia: pathophysiology, challenges, and perspectives. Circ Res. 2019;124:1094–112. 10.1161/CIRCRESAHA.118.313276 30920918

[8] Lunghi L, Ferretti ME, Medici S, Biondi C, Vesce F. Control of human trophoblast function. Reprod Biol Endocrinol. 2007;5:6. 10.1186/1477-7827-5-6 PMC1800852 17288592

[9] Li Y, Yan J, Chang H-M, Chen Z-J, Leung PCK. Roles of TGF-β superfamily proteins in extravillous trophoblast invasion. Trends Endocrinol Metab. 2021;32:170–89. 10.1016/j.tem.2020.12.005 33478870

[10] Aluganti Narasimhulu C, Singla DK. The role of bone morphogenetic protein 7 (BMP-7) in inflammation in heart diseases. Cells. 2020;9:280. 10.3390/cells9020280 PMC7073173 31979268

[11] Monsivais D, Clementi C, Peng J, Fullerton PT, Prunskaite-Hyyryläinen R, Vainio SJ, et al. BMP7 induces uterine receptivity and blastocyst attachment. Endocrinology. 2017;158:979–92. 10.1210/en.2016-1629 PMC5460793 28324064

[12] Yuan C, Li X, Song H, Fan L, Su S, Dong B. BMP7 coordinates endometrial epithelial cell receptivity for blastocyst implantation through the endoglin pathway in cell lines and a mouse model. Exp Ther Med. 2019;17:2547–56. 10.3892/etm.2019.7265 PMC6425146 30906444

[13] Singla DK, Singla R, Wang J. BMP-7 treatment increases M2 macrophage differentiation and reduces inflammation and plaque formation in Apo E-/- mice. PLoS ONE. 2016;11:e0147897. 10.1371/journal.pone.0147897 PMC4732822 26824441

[14] Rocher C, Singla R, Singal PK, Parthasarathy S, Singla DK. Bone morphogenetic protein 7 polarizes THP-1 cells into M2 macrophages. Can J Physiol Pharm. 2012;90:947–51. 10.1139/y2012-102 22720873

[15] Staff AC, Dechend R, Pijnenborg R. Learning from the placenta. Hypertension. 2010;56:1026–34. 10.1161/HYPERTENSIONAHA.110.157743 20956732

[16] Tatsukawa H, Hitomi K. Role of transglutaminase 2 in cell death, survival, and fibrosis. Cells. 2021;10:1842. 10.3390/cells10071842 PMC8307792 34360011

[17] Yao Z, Fan Y, Lin L, Kellems RE, Xia Y. Tissue transglutaminase: a multifunctional and multisite regulator in health and disease. Physiol Rev. 2024;104:281–325. 10.1152/physrev.00003.2023 37712623

[18] Liu C, Wang W, Parchim N, Irani RA, Blackwell SC, Sibai B, et al. Tissue transglutaminase contributes to the pathogenesis of preeclampsia and stabilizes placental angiotensin receptor type 1 by ubiquitination-preventing isopeptide modification. Hypertension. 2014;63:353–61. 10.1161/HYPERTENSIONAHA.113.02361 PMC4052572 24191290

[19] Welge-Lüssen U, May CA, Lütjen-Drecoll E. Induction of tissue transglutaminase in the trabecular meshwork by TGF-beta1 and TGF-beta2. Invest Ophthalmol Vis Sci. 2000;41:2229–38. 10892867

[20] National Research Council, Division on Earth, Life Studies, Institute for Laboratory Animal Research, Committee for the Update of the Guide for the Care, Use of Laboratory Animals. Guide for the Care and Use of Laboratory Animals, 8th edn. National Academies Press: Washington, DC, 2011.

[21] Lu F, Longo M, Tamayo E, Maner W, Al-Hendy A, Anderson GD, et al. The effect of over-expression of sFlt-1 on blood pressure and the occurrence of other manifestations of preeclampsia in unrestrained conscious pregnant mice. Am J Obstet Gynecol. 2007;196:396.e1–396.e7. 10.1016/j.ajog.2006.12.024 17403433

[22] Zhang Q, Lee C-L, Yang T, Li J, Zeng Q, Liu X, et al. Adrenomedullin has a pivotal role in trophoblast differentiation: a promising nanotechnology-based therapeutic target for early-onset preeclampsia. Sci Adv. 2023;9:eadi4777. 10.1126/sciadv.adi4777 PMC10624351 37922358

[23] Deng J, Zhao H-J, Zhong Y, Hu C, Meng J, Wang C, et al. H3K27me3-modulated Hofbauer cell BMP2 signalling enhancement compensates for shallow trophoblast invasion in preeclampsia. eBioMedicine 2023;93:104664. 10.1016/j.ebiom.2023.104664 PMC10300321 37331163

[24] Vento-Tormo R, Efremova M, Botting RA, Turco MY, Vento-Tormo M, Meyer KB, et al. Single-cell reconstruction of the early maternal–fetal interface in humans. Nature. 2018;563:347–53. 10.1038/s41586-018-0698-6 PMC7612850 30429548

[25] Zhang T, Bian Q, Chen Y, Wang X, Yu S, Liu S, et al. Dissecting human trophoblast cell transcriptional heterogeneity in preeclampsia using single-cell RNA sequencing. Mol Genet Genom Med. 2021;9:e1730. 10.1002/mgg3.1730 PMC8404237 34212522

[26] Zhou W, Wang H, Yang Y, Guo F, Yu B, Su Z. Trophoblast cell subtypes and dysfunction in the placenta of individuals with preeclampsia revealed by single‑cell RNA sequencing. Mol Cells. 2022;45:317–28. 10.14348/molcells.2021.0211 PMC9095508 35289305

[27] Zhu S, Li Z, Cui L, Ban Y, Leung PCK, Li Y, et al. Activin A increases human trophoblast invasion by upregulating integrin β1 through ALK4. FASEB J 2021;35:e21735. 10.1096/fj.202001604R PMC12315494 33230889

[28] Sheridan MA, Fernando RC, Gardner L, Hollinshead MS, Burton GJ, Moffett A, et al. Establishment and differentiation of long-term trophoblast organoid cultures from the human placenta. Nat Protoc. 2020;15:3441–63. 10.1038/s41596-020-0381-x 32908314

[29] Lyu C, Ni T, Guo Y, Zhou T, Chen Z, Yan J, et al. Insufficient GDF15 expression predisposes women to unexplained recurrent pregnancy loss by impairing extravillous trophoblast invasion. Cell Prolif. 2023;56:e13514. 10.1111/cpr.13514 PMC10693185 37272232

[30] Zhao H-J, Klausen C, Zhu H, Chang H-M, Li Y, Leung PCK. Bone morphogenetic protein 2 promotes human trophoblast cell invasion and endothelial-like tube formation through ID1-mediated upregulation of IGF binding protein-3. FASEB J Publ Fed Am Soc Exp Biol. 2020;34:3151–64. 10.1096/fj.201902168RR 31908038

[31] Yi Y, Zhu H, Klausen C, Leung PCK. Transcription factor SOX4 facilitates BMP2-regulated gene expression during invasive trophoblast differentiation. FASEB J. 2021;35:e22028. 10.1096/fj.202100925RR 34739154

[32] Li F, Ssengonzi R, Townley-Tilson WD, Kayashima Y, Maeda-Smithies N. 332 IGF-1 mediates ID2 in trophoblast differentiation. Am J Obstet Gynecol. 2024;230:S188–9.

[33] Lan X, Guo L, Hu C, Zhang Q, Deng J, Wang Y, et al. Fibronectin mediates activin A-promoted human trophoblast migration and acquisition of endothelial-like phenotype. Cell Commun Signal. 2024;22:61. 10.1186/s12964-023-01463-z PMC10807102 38263146

[34] Fisher SJ. Why is placentation abnormal in preeclampsia? Am J Obstet Gynecol. 2015;213:S115–122. 10.1016/j.ajog.2015.08.042 PMC4592742 26428489

[35] Zhang J, Jing S, Zhang H, Zhang J, Xie H, Feng L. Low-dose aspirin prevents LPS-induced preeclampsia-like phenotype via AQP-1 and the MAPK/ERK 1/2 pathway. Placenta. 2022;121:61–69. 10.1016/j.placenta.2022.03.007 35279428

[36] Haider S, Lackner AI, Dietrich B, Kunihs V, Haslinger P, Meinhardt G, et al. Transforming growth factor-β signaling governs the differentiation program of extravillous trophoblasts in the developing human placenta. Proc Natl Acad Sci USA 2022;119:e2120667119. 10.1073/pnas.2120667119 PMC9282384 35867736

[37] Zhao H, Klausen C, Zhu H, Chang H, Li Y, Leung PCK. Bone morphogenetic protein 2 promotes human trophoblast cell invasion and endothelial-like tube formation through ID1-mediated upregulation of IGF binding protein-3. FASEB J. 2020;34:3151–64. 10.1096/fj.201902168RR 31908038

[38] Wu Z, Fang L, Yang S, Gao Y, Wang Z, Meng Q, et al. GDF-11 promotes human trophoblast cell invasion by increasing ID2-mediated MMP2 expression. Cell Commun Signal CCS. 2022;20:89. 10.1186/s12964-022-00899-z PMC9202197 35705978

[39] Liu Z, Wang C, Pei J, Li M, Gu W. SIRT1: a novel protective molecule in pre-eclampsia. Int J Med Sci. 2022;19:993. 10.7150/ijms.73012 PMC9254375 35813294

[40] Coxam B, Collins RT, Hußmann M, Huisman Y, Meier K, Jung S, et al. Svep1 stabilises developmental vascular anastomosis in reduced flow conditions. Development. 2022;149:dev199858. 10.1242/dev.199858 PMC8977097 35312765

[41] Mead TJ. ADAMTS6: Emerging roles in cardiovascular, musculoskeletal and cancer biology. Front Mol Biosci. 2022;9:1023511. 10.3389/fmolb.2022.1023511 PMC9627330 36339722

[42] Leach RE, Romero R, Kim YM, Chaiworapongsa T, Kilburn B, Das SK, et al. Pre-eclampsia and expression of heparin-binding EGF-like growth factor. Lancet. 2002;360:1215–9. 10.1016/S0140-6736(02)11283-9 12401248

[43] Zhang Y, Le T, Grabau R, Mohseni Z, Kim H, Natale DR, et al. TMEM16F phospholipid scramblase mediates trophoblast fusion and placental development. Sci Adv. 2020;6:eaba0310. 10.1126/sciadv.aba0310 PMC7202889 32494719

[44] Masum MA, Ichii O, Elewa YHA, Nakamura T, Kon Y. Local CD34-positive capillaries decrease in mouse models of kidney disease associating with the severity of glomerular and tubulointerstitial lesions. BMC Nephrol. 2017;18:280. 10.1186/s12882-017-0694-3 PMC5584339 28870174

[45] Kusano T, Takano H, Kang D, Nagahama K, Aoki M, Morita M, et al. Endothelial cell injury in acute and chronic glomerular lesions in patients with IgA nephropathy. Hum Pathol. 2016;49:135–44. 10.1016/j.humpath.2015.10.013 26826420

[46] Guan H, Li J, Sun R, Liu W, Feng M, Ma H, et al. Expression of BMP7 In ovarian cancer and biological effect of BMP7 knockdown on ovarian cancer cells. OncoTargets Ther. 2019;12:7897–909. 10.2147/OTT.S217975 PMC6769165 31576147

[47] Sinnberg T, Niessner H, Levesque MP, Dettweiler C, Garbe C, Busch C. Embryonic bone morphogenetic protein and nodal induce invasion in melanocytes and melanoma cells. Biol Open. 2018;7:bio032656. 10.1242/bio.032656 PMC6031345 29716947

[48] Hager H, Gliemann J, Hamilton-Dutoit S, Ebbesen P, Koppelhus U, Henning Jensen P. Developmental regulation of tissue transglutaminase during human placentation and expression in neoplastic trophoblast. J Pathol. 1997;181:106–10. 10.1002/(SICI)1096-9896(199701)181:1<106::AID-PATH725>3.0.CO;2-K 9072011

[49] Akimov SS, Belkin AM. Cell-surface transglutaminase promotes fibronectin assembly via interaction with the gelatin-binding domain of fibronectin: a role in TGFβ-dependent matrix deposition. J Cell Sci. 2001;114:2989–3000. 10.1242/jcs.114.16.2989 11686302

[50] Cheng J-C, Chang H-M, Leung PCK. Transforming growth factor-β1 inhibits trophoblast cell invasion by inducing snail-mediated down-regulation of vascular endothelial-cadherin protein. J Biol Chem. 2013;288:33181–92. 10.1074/jbc.M113.488866 PMC3829165 24106276

[51] Muy-Rivera M. Transforming growth factor-β1 (TGF-β1) in plasma is associated with preeclampsia risk in Peruvian women with systemic inflammation. Am J Hypertens. 2004;17:334–8. 10.1016/j.amjhyper.2003.12.010 15062887

[52] Benian A, Madazli R, Aksu F, Uzun H, Aydin S. Plasma and placental levels of interleukin-10, transforming growth factor-beta1, and epithelial-cadherin in preeclampsia. Obstet Gynecol. 2002;100:327–31. 10.1016/s0029-7844(02)02077-x 12151158

[53] Ohmaru-Nakanishi T, Asanoma K, Fujikawa M, Fujita Y, Yagi H, Onoyama I, et al. Fibrosis in preeclamptic placentas is associated with stromal fibroblasts activated by the transforming growth factor-β1 signaling pathway. Am J Pathol. 2018;188:683–95. 10.1016/j.ajpath.2017.11.008 29253459

[54] Boon MR, Van Der Horst G, Van Der Pluijm G, Tamsma JT, Smit JWA, Rensen PCN. Bone morphogenetic protein 7: a broad-spectrum growth factor with multiple target therapeutic potency. Cytokine Growth Factor Rev. 2011;22:221–9. 10.1016/j.cytogfr.2011.08.001 21924665

[55] Ning J, Ye Y, Bu D, Zhao G, Song T, Liu P, et al. Imbalance of TGF-β1/BMP-7 pathways induced by M2-polarized macrophages promotes hepatocellular carcinoma aggressiveness. Mol Ther J Am Soc Gene Ther. 2021;29:2067–87. 10.1016/j.ymthe.2021.02.016 PMC8178441 33601054

[56] Zhao H-J, Chang H-M, Zhu H, Klausen C, Li Y, Leung PCK. Bone morphogenetic protein 2 promotes human trophoblast cell invasion by inducing activin A production. Endocrinology. 2018;159:2815–25. 10.1210/en.2018-00301 29846546

[57] King A, Ndifon C, Lui S, Widdows K, Kotamraju VR, Agemy L, et al. Tumor-homing peptides as tools for targeted delivery of payloads to the placenta. Sci Adv. 2016;2:e1600349. 10.1126/sciadv.1600349 PMC4928982 27386551

