# Supplementary material for: Role of the trophoblastic BMP7-TGM2 axis in the pathogenesis and prevention of preeclampsia
Source: Cell Death Dis. 2026 Jul 11;17(1):865. doi: 10.1038/s41419-026-09111-3 (PMC13642358; doi:10.1038/s41419-026-09111-3)

Figure 3C

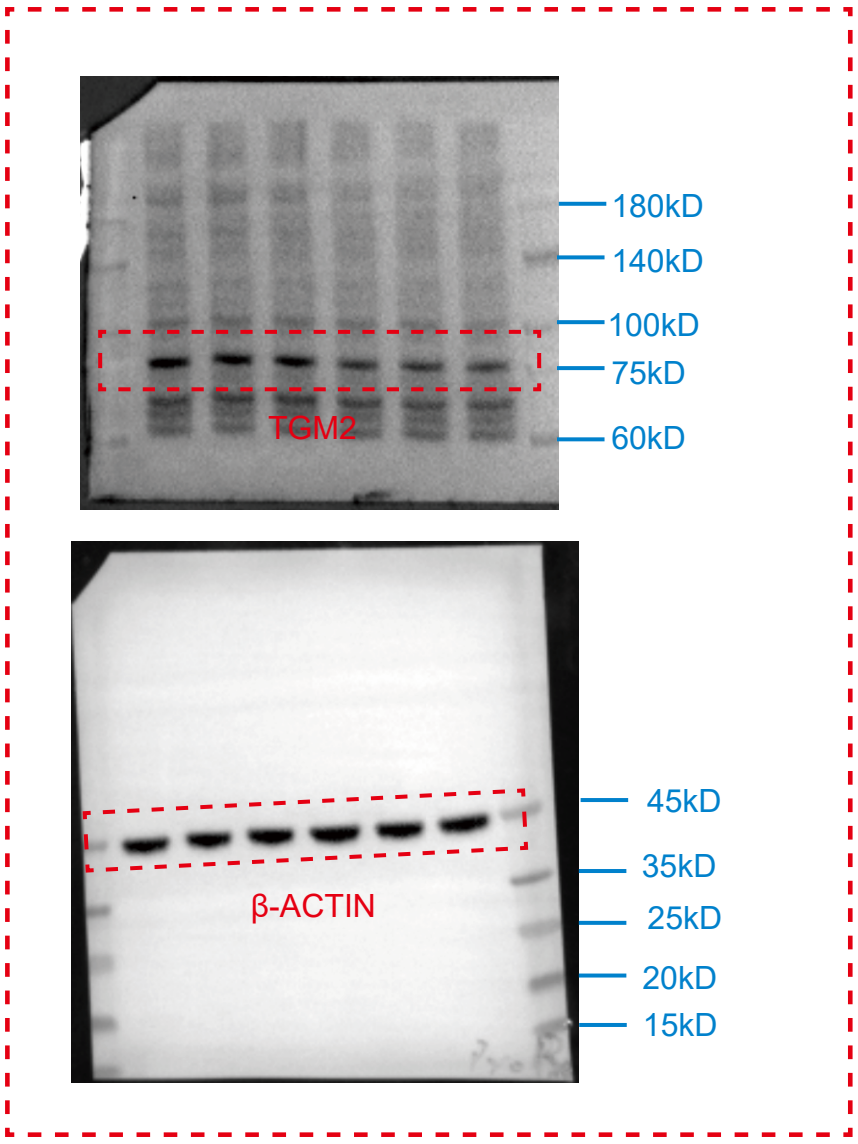

Figure 4B

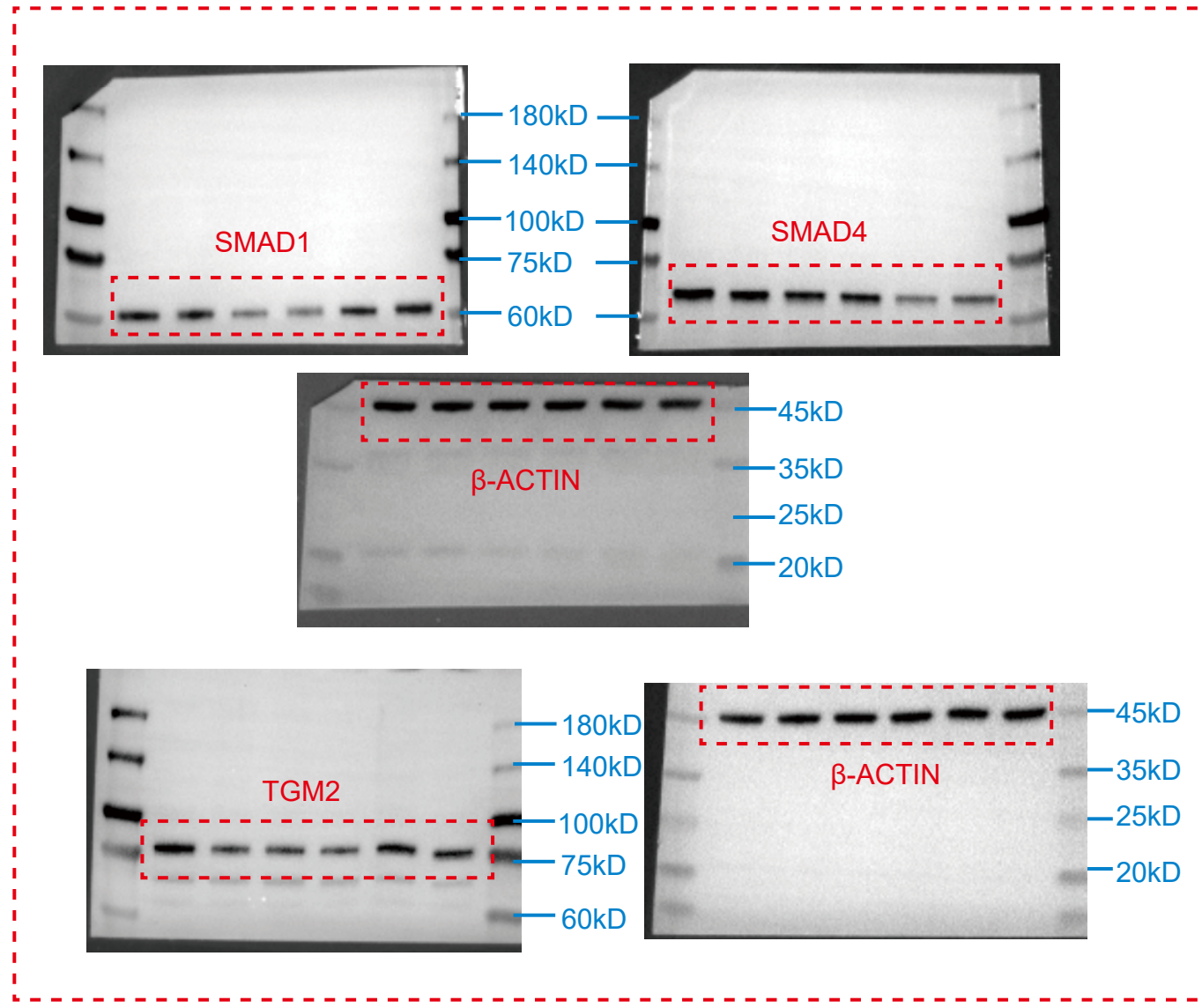

Figure 4A

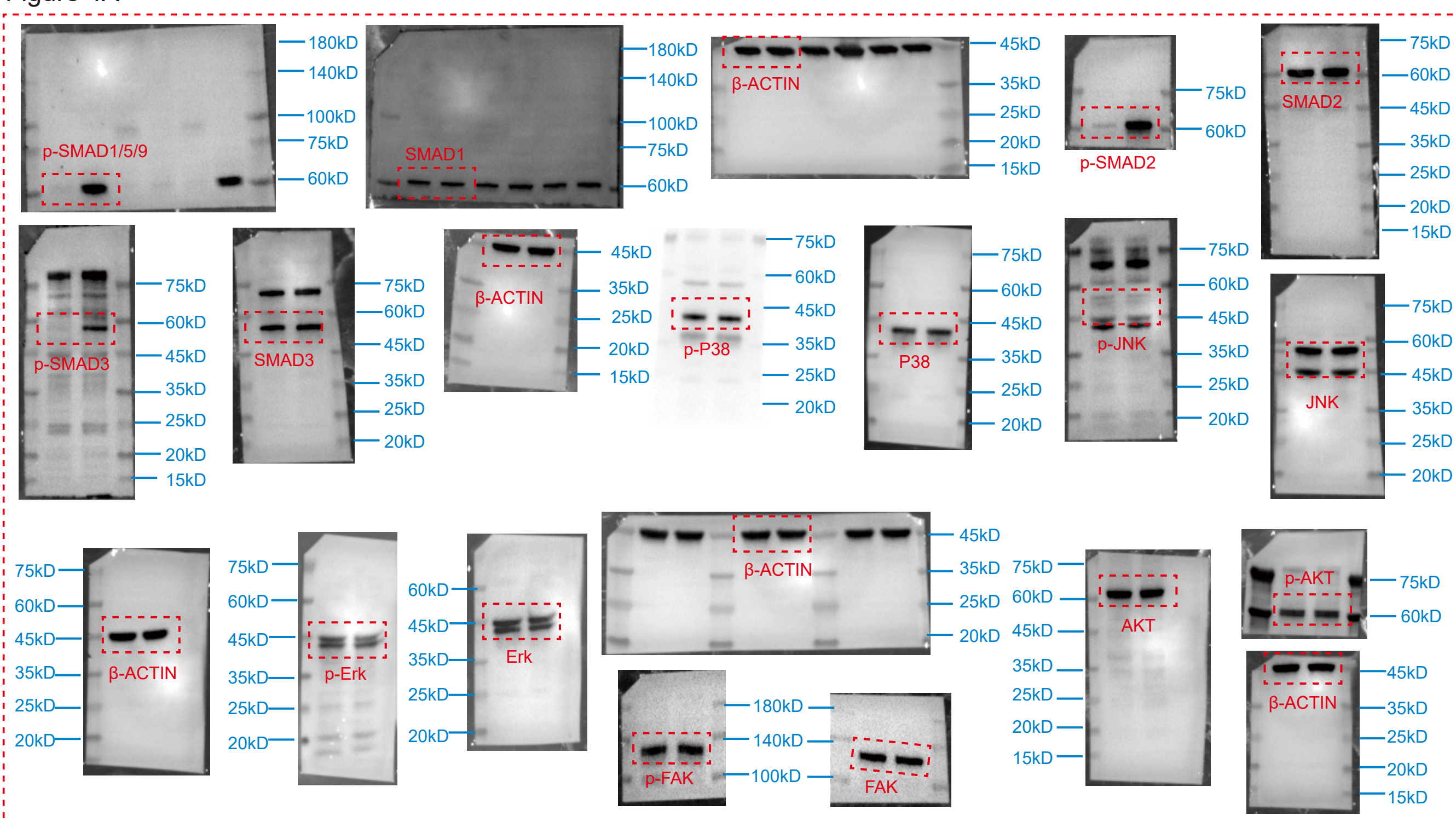

Figure 4C

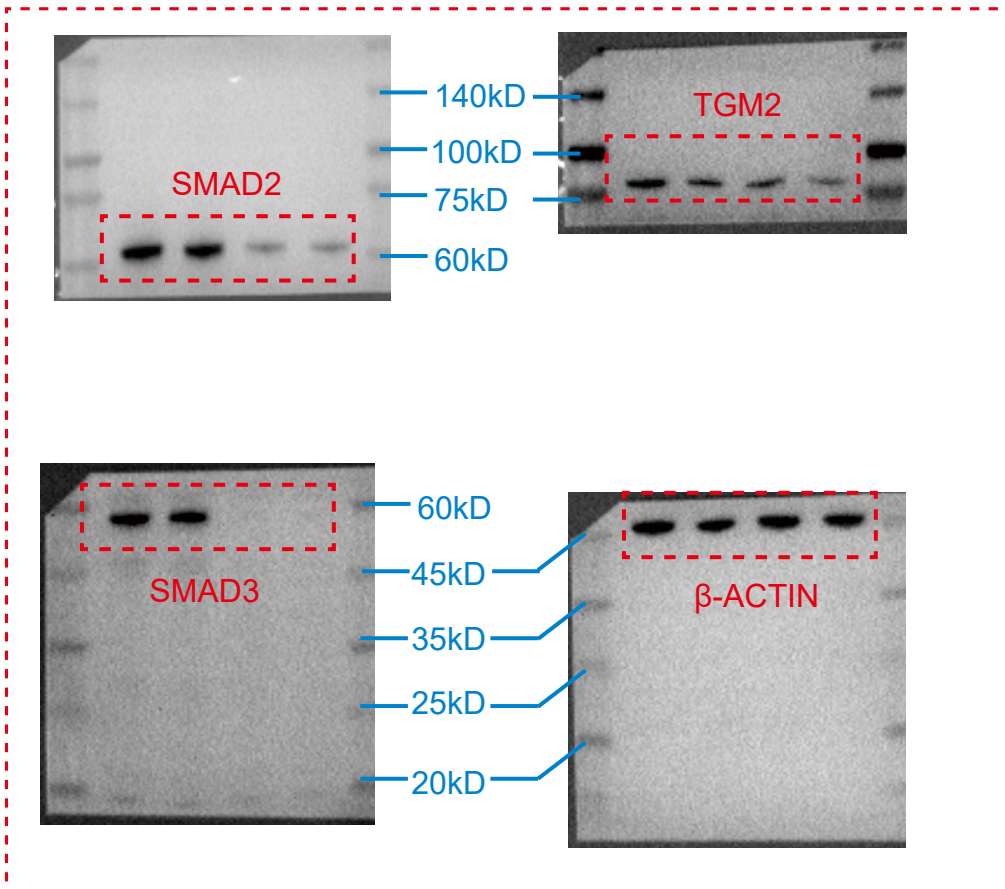

Figure 4D

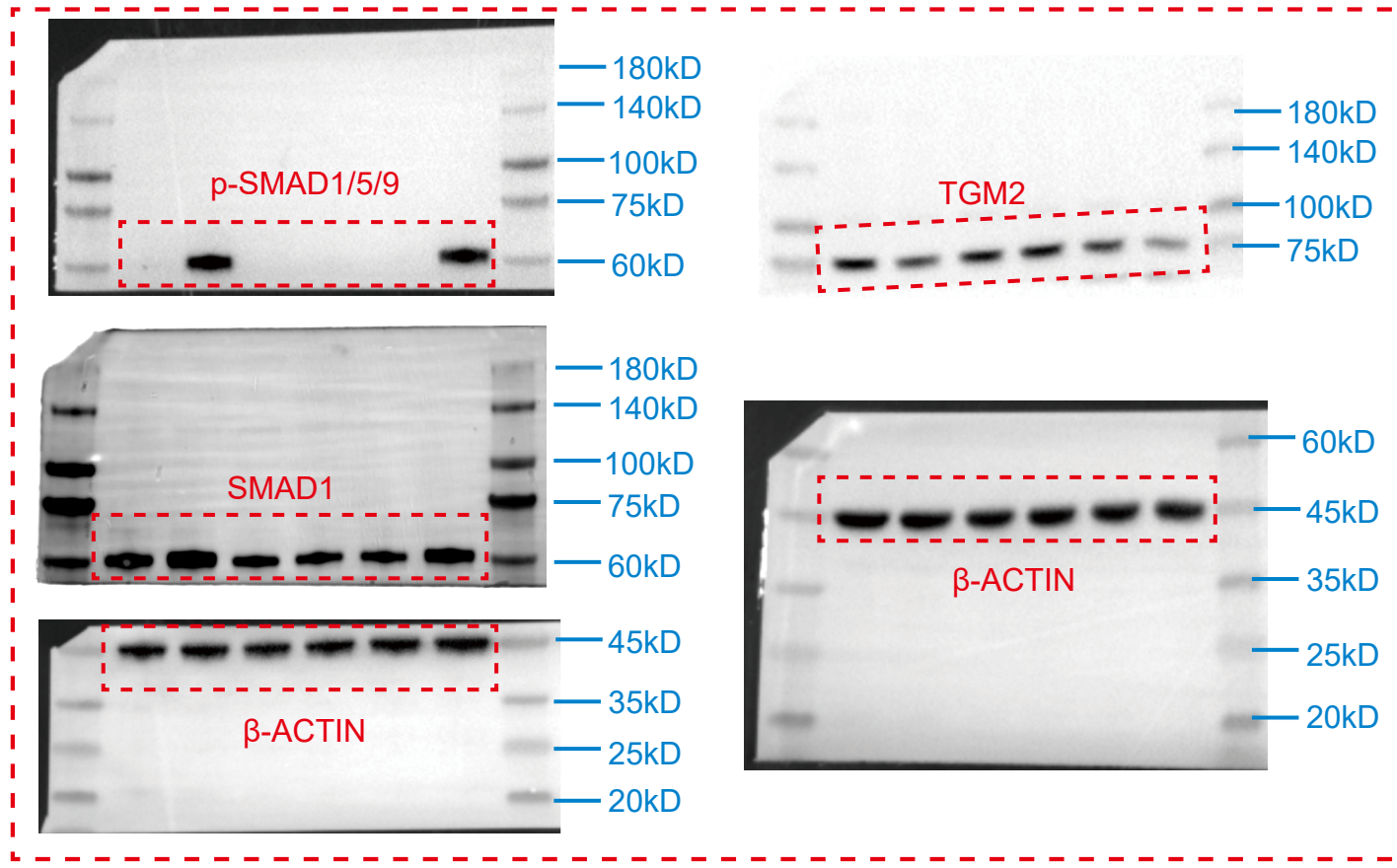

Figure 4E

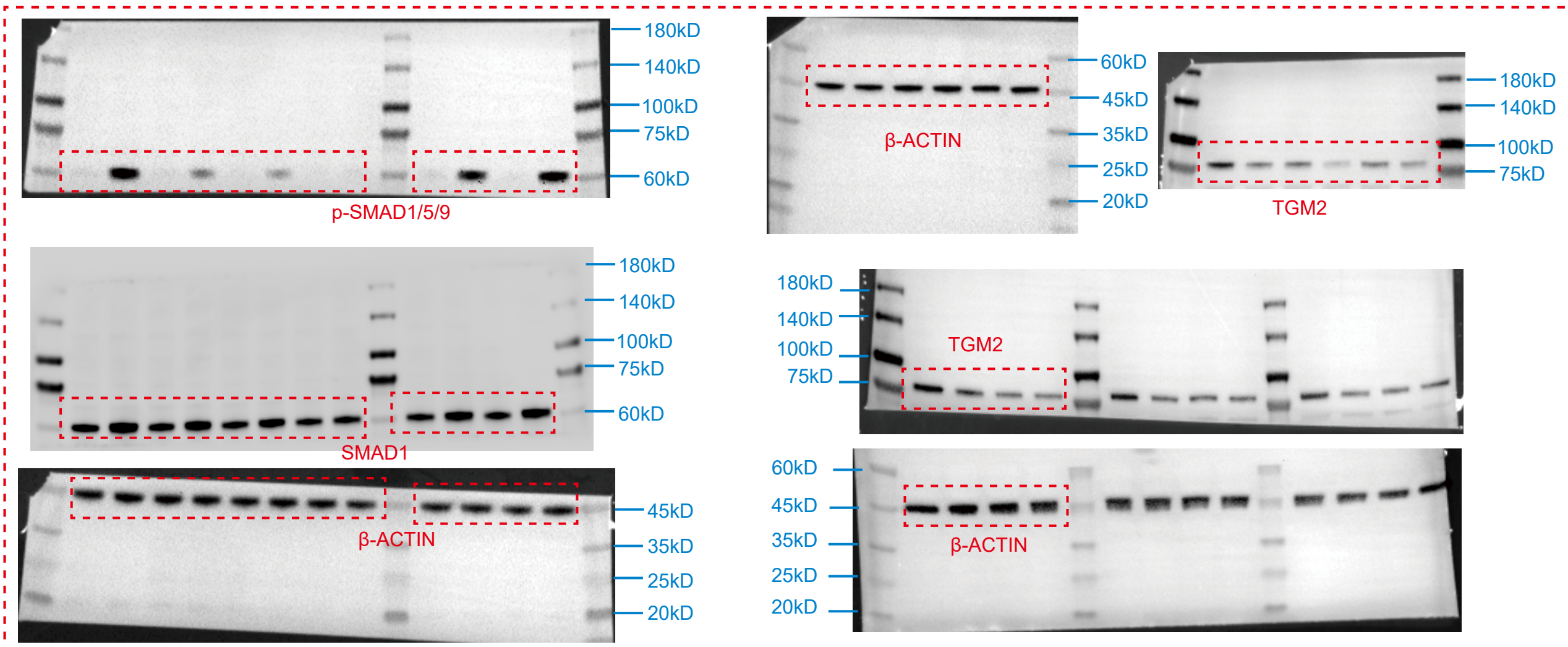

Figure 5B

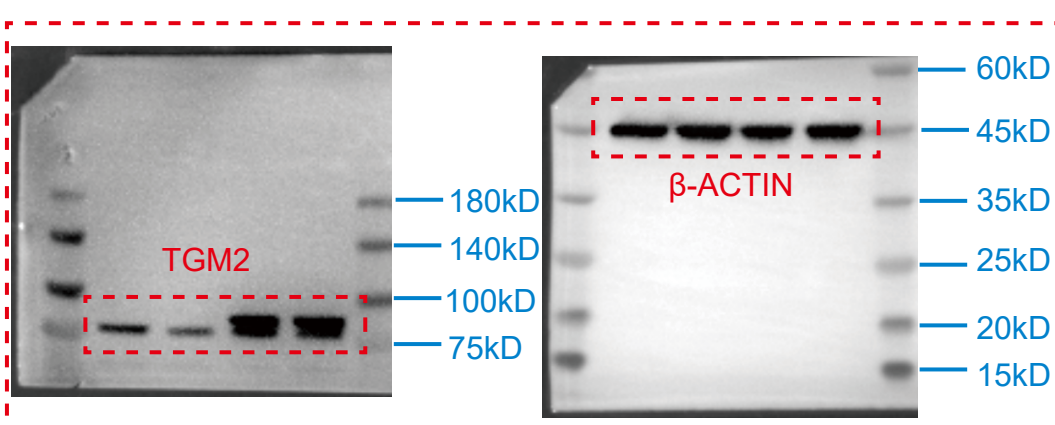

Figure 5F

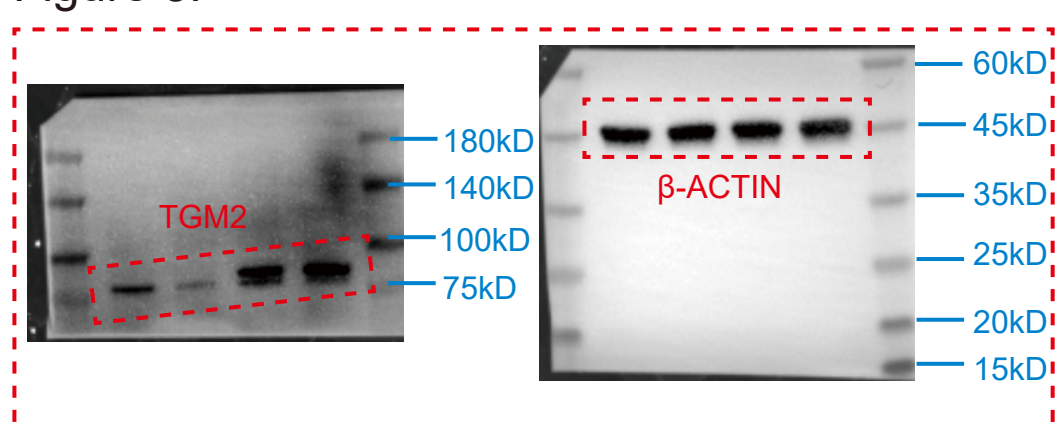

Figure S1A

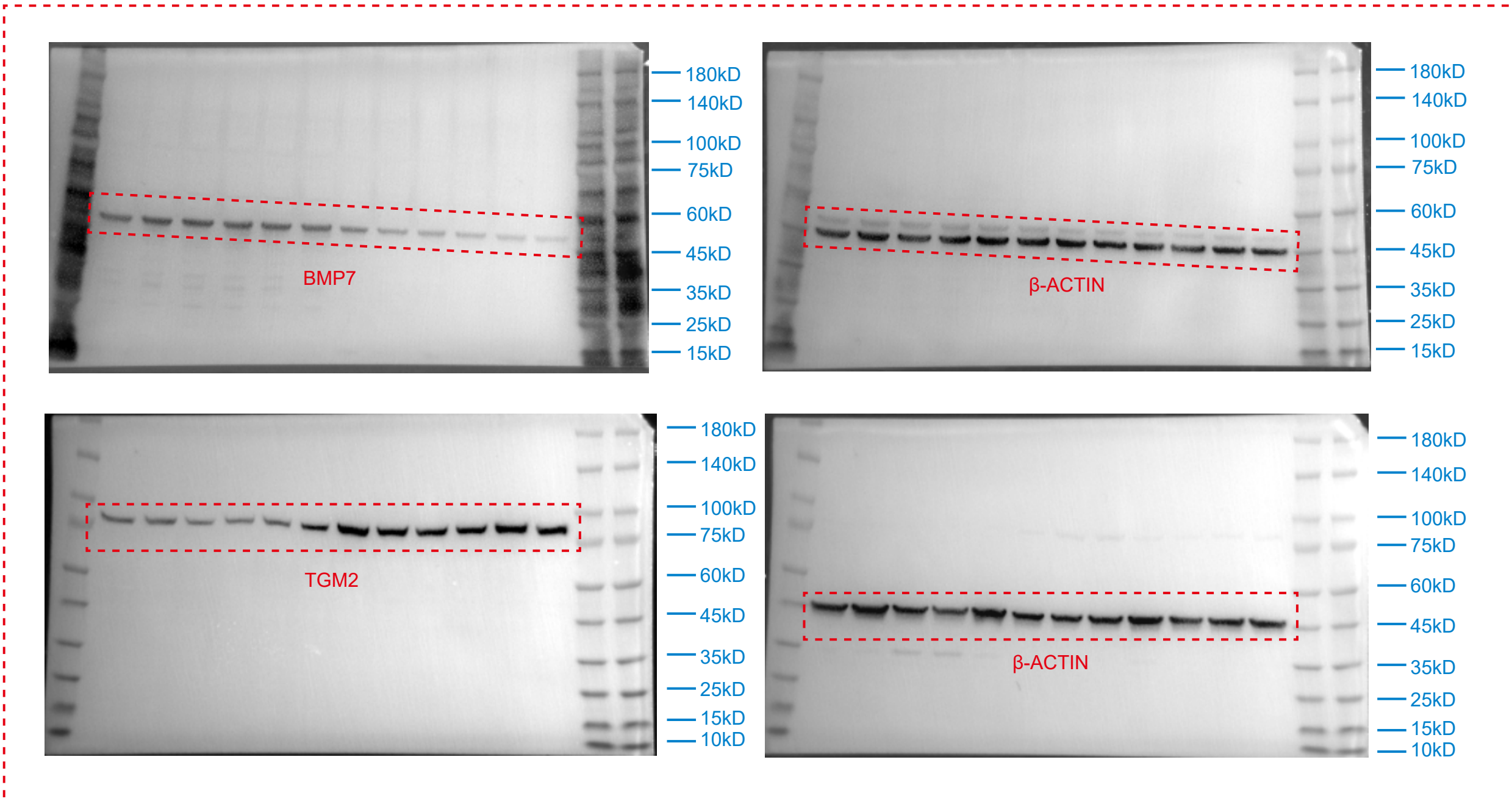
180kD
140kD
100kD
75kD
60kD
45kD
BMP7
35kD
25kD
15kD
180kD
140kD
100kD
75kD
60kD
45kD
β-ACTIN
35kD
25kD
15kD
180kD
140kD
100kD
75kD
TGM2
60kD
45kD
35kD
25kD
15kD
10kD
180kD
140kD
100kD
75kD
60kD
45kD
β-ACTIN
35kD
25kD
15kD
10kD

Figure S2A

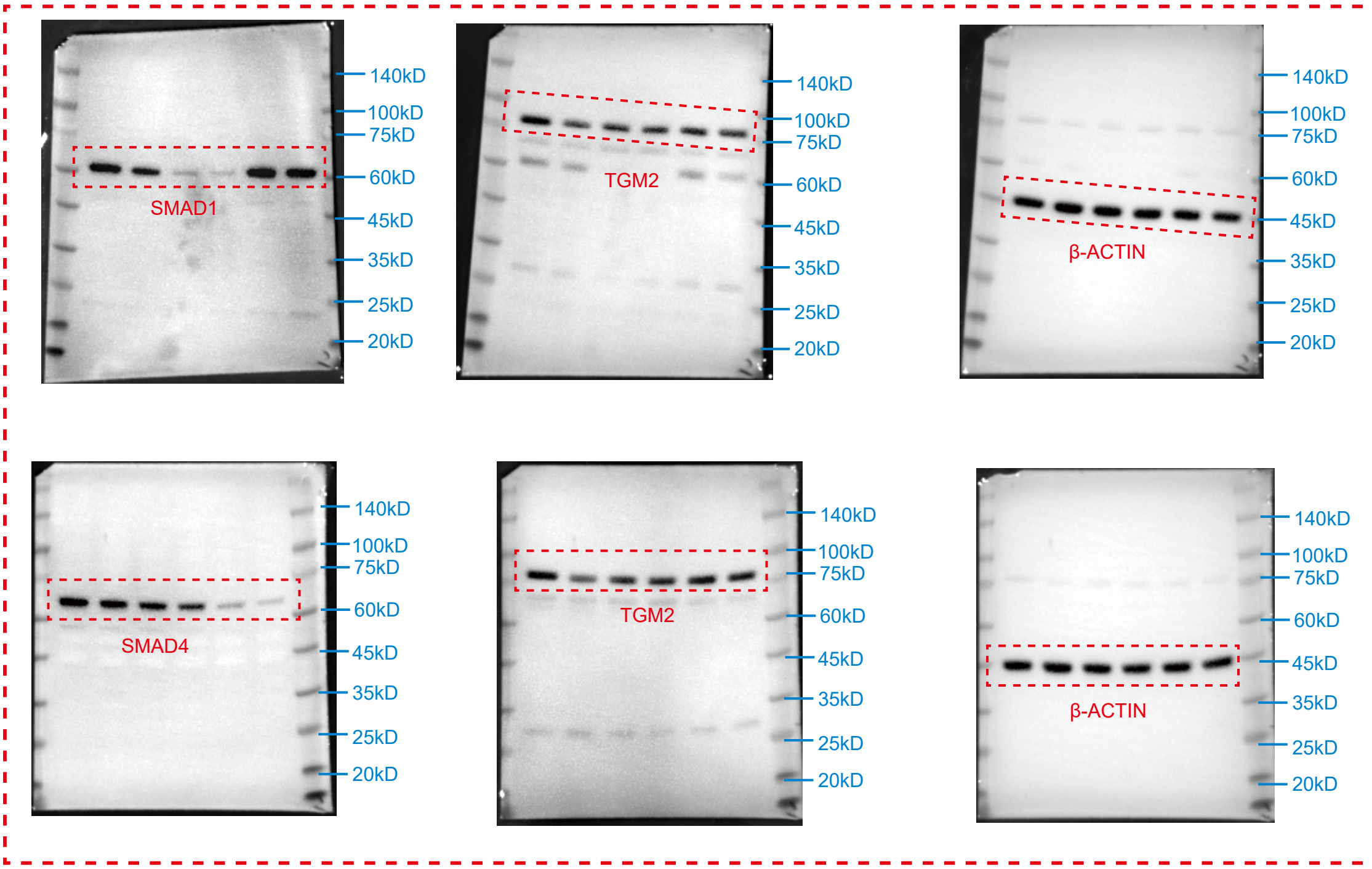

Figure S2B

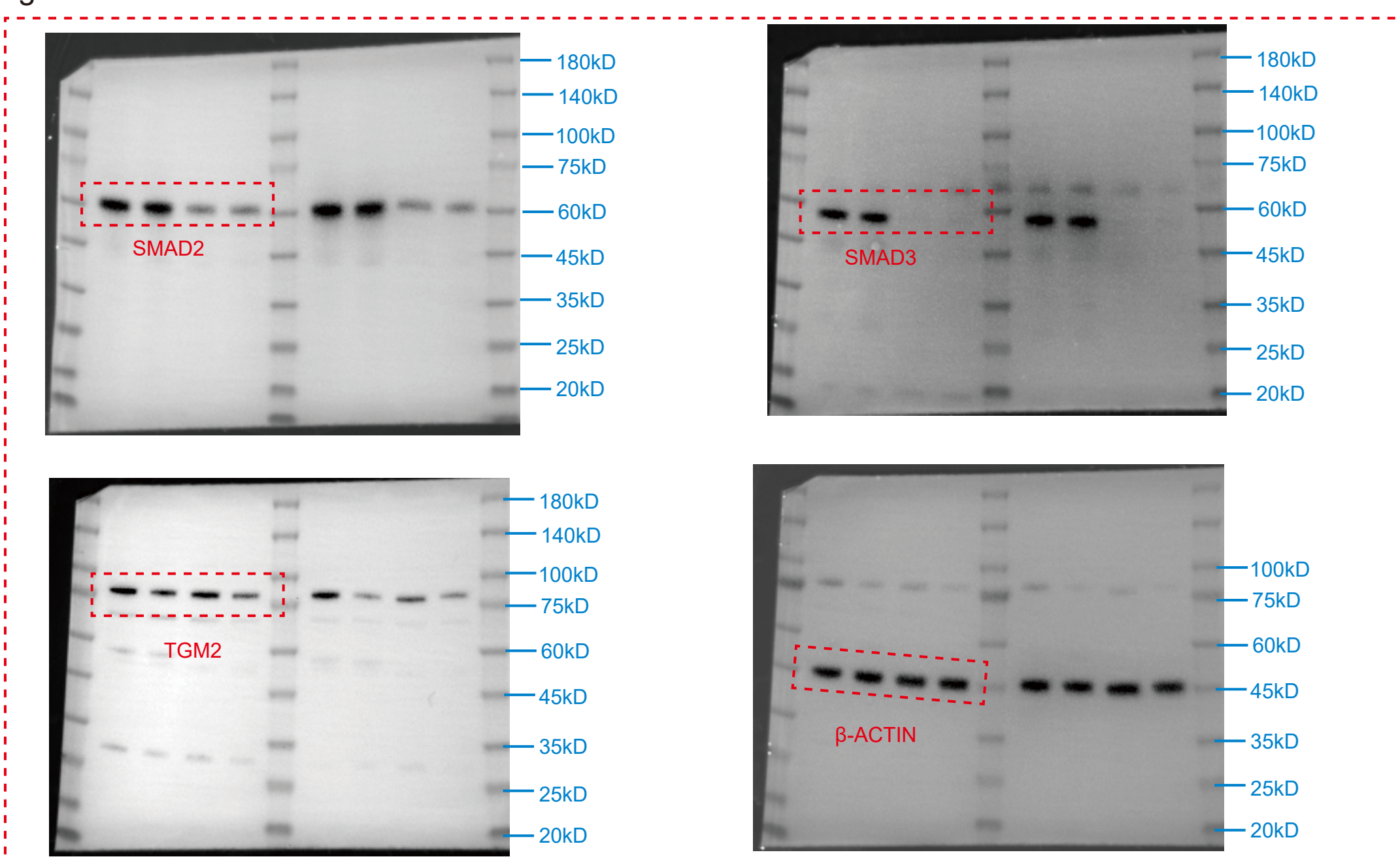

Figure S2C

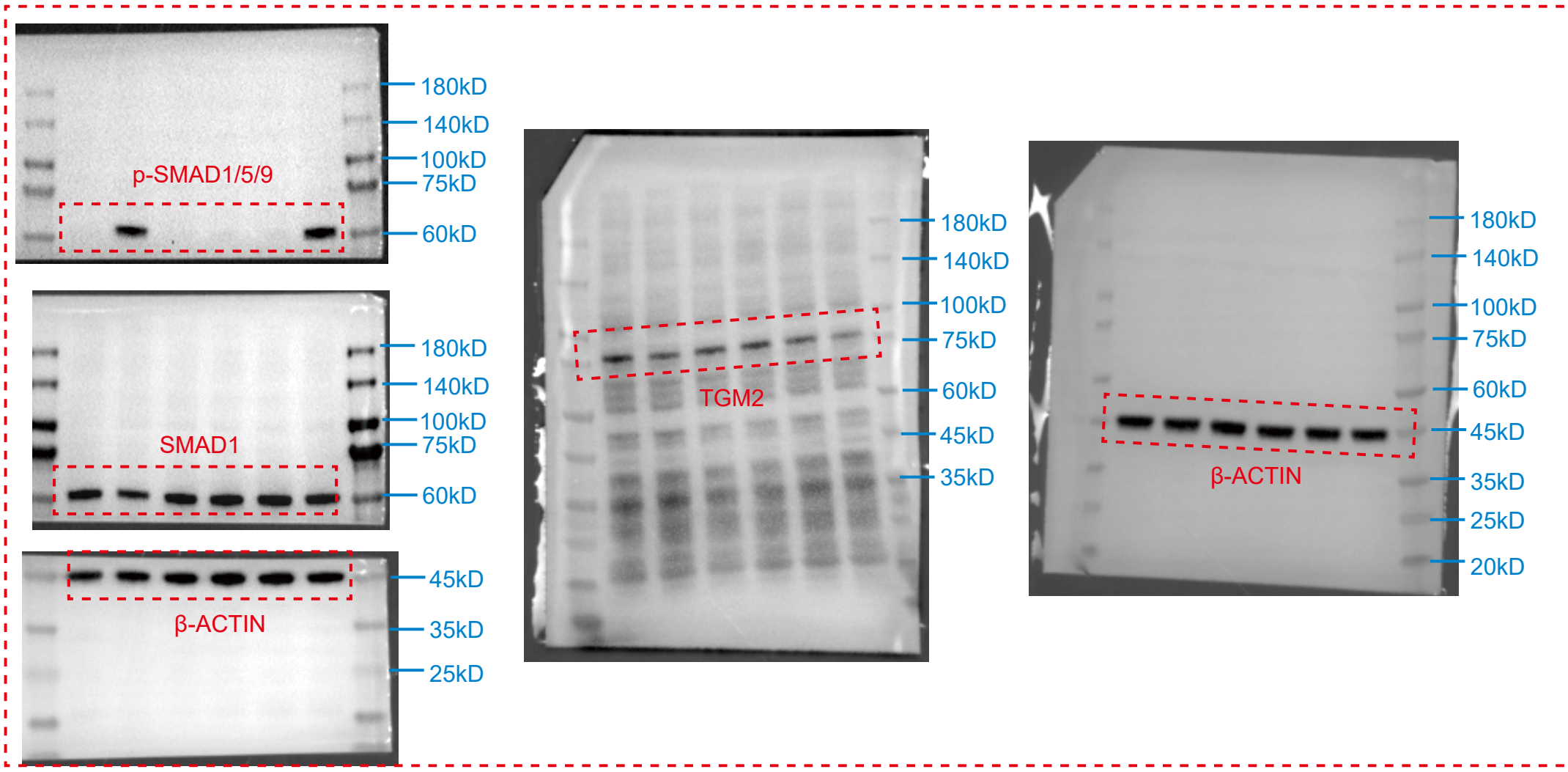

Figure S2D

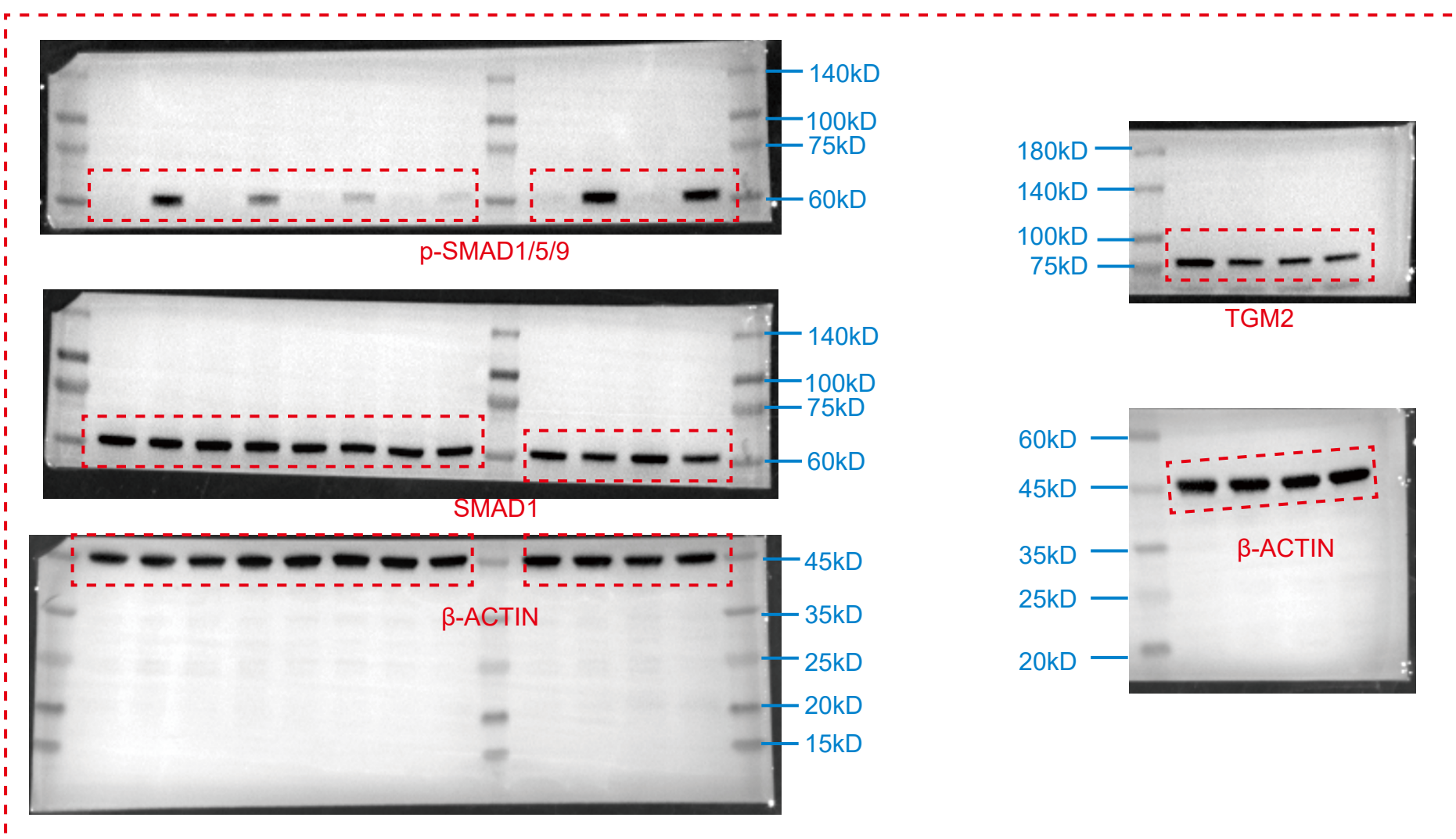

Figure S5B

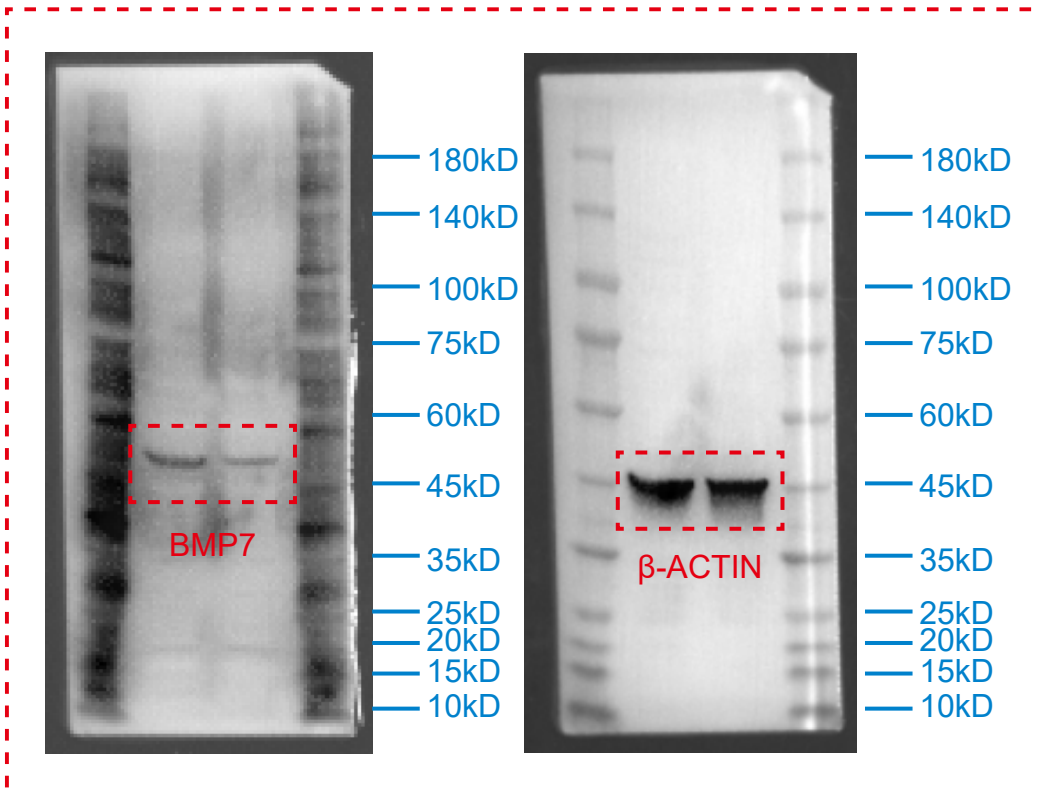

Figure S5D

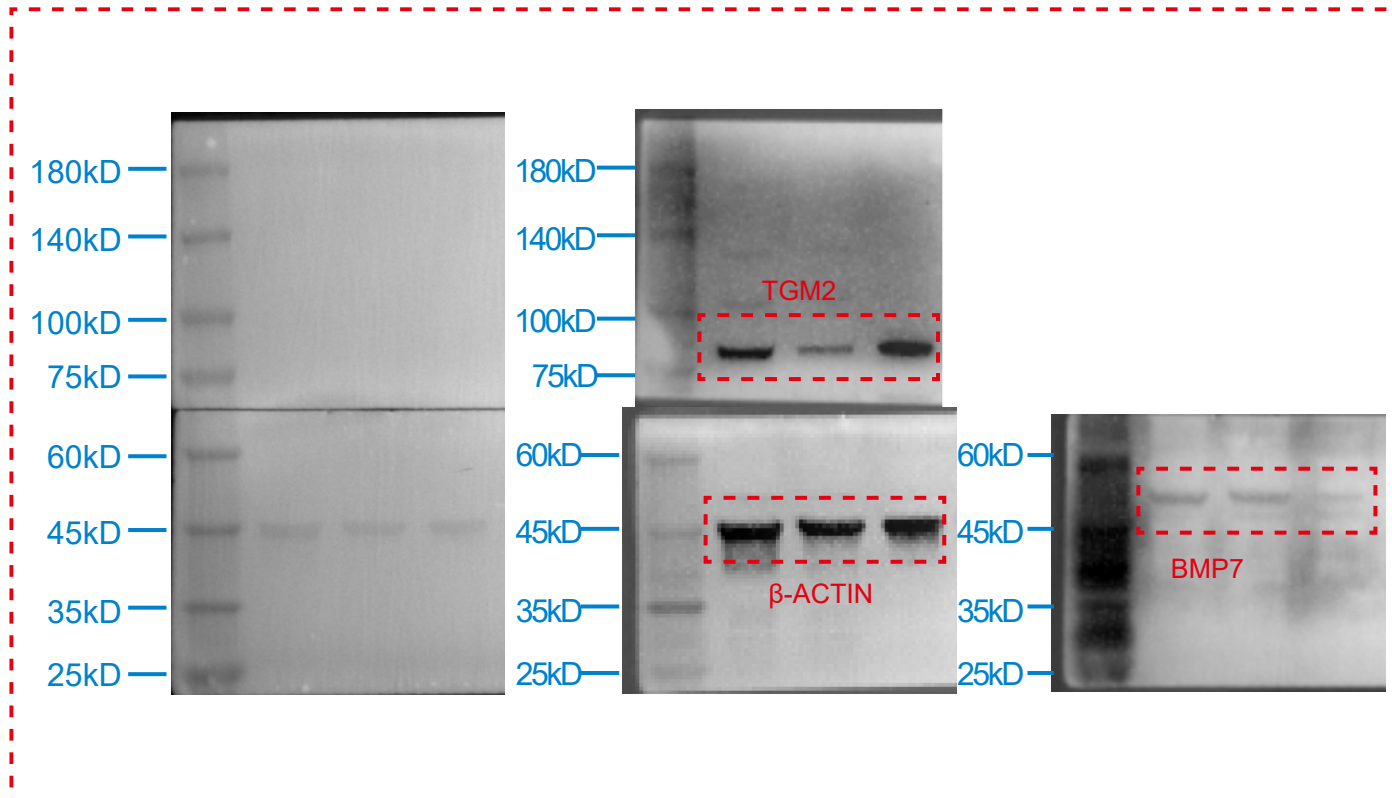

Figure S5H

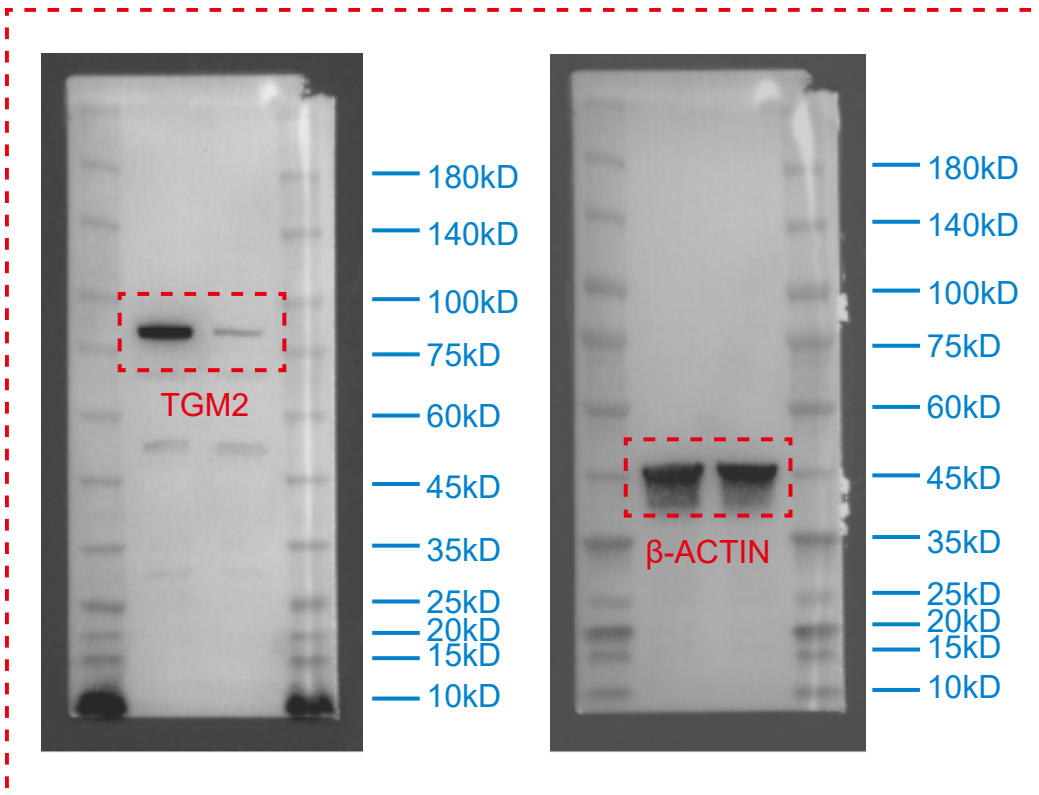

Figure S5J

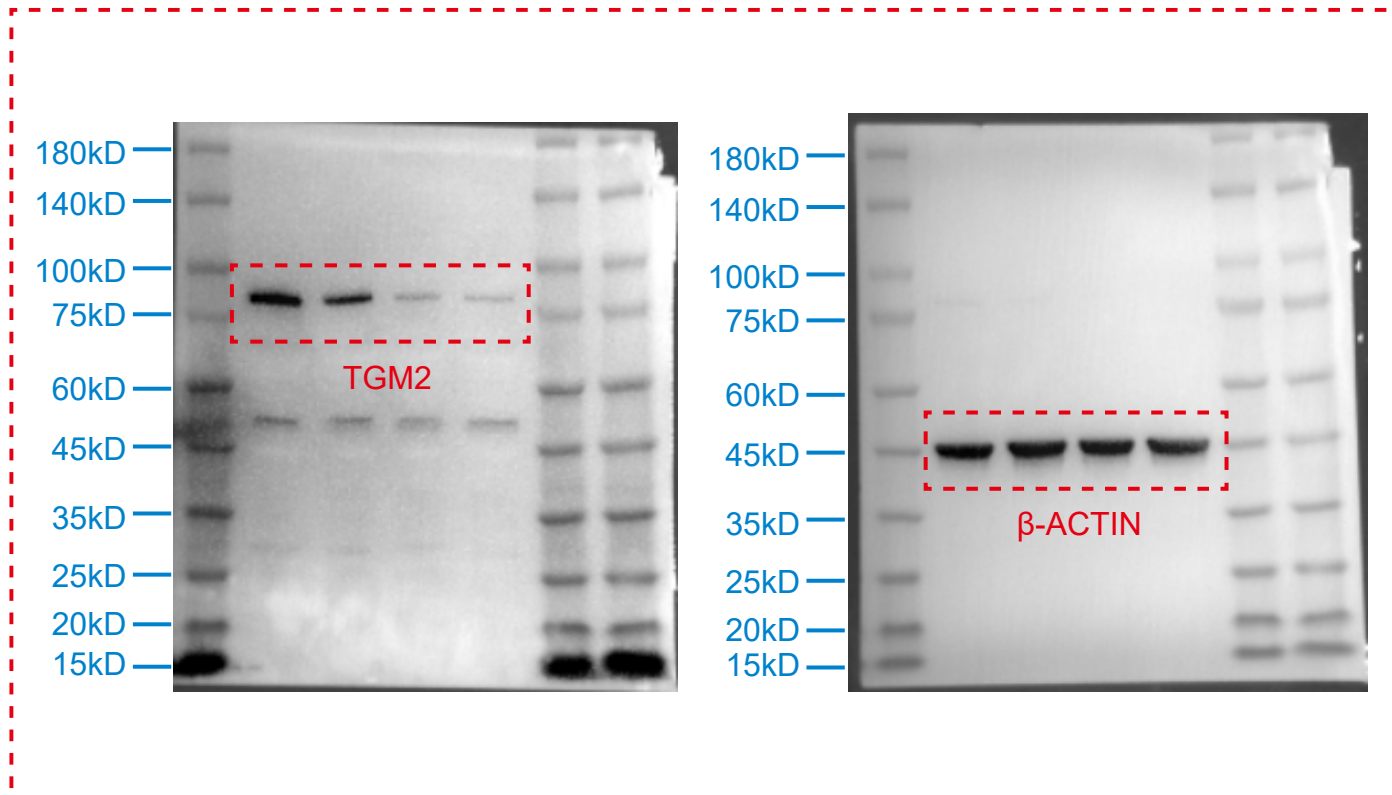

Supplement: Supplementary file 1 [file 41419_2026_9111_MOESM2_ESM.pdf]
