# Supplementary material for: Role of the trophoblastic BMP7-TGM2 axis in the pathogenesis and prevention of preeclampsia
Source: Cell Death Dis. 2026 Jul 11;17(1):865. doi: 10.1038/s41419-026-09111-3 (PMC13642358; doi:10.1038/s41419-026-09111-3)
Supplement: Supplementary file 2 [file 41419_2026_9111_MOESM1_ESM.docx]

**Supplementary Information**

**For**

**Role of the trophoblastic BMP7-TGM2 axis in the pathogenesis and prevention of preeclampsia**

Qiaoqiao Ding, Mingxi Liu, Binbin Zhao, Yaxin Su, Ying Zhong, Cameron Klein, Junhao Yan, Peter C.K. Leung, Zi-Jiang Chen, Daimin Wei, Yan Li

Correspondence to:

Yan Li, MD, PhD, State Key Laboratory of Reproductive Medicine and Offspring Health, Shandong University, Jinan, Shandong, China. E-mail: [ubcliyan@sdu.edu.cn](mailto:ubcliyan@sdu.edu.cn)

Daimin Wei, MD, PhD, State Key Laboratory of Reproductive Medicine and Offspring Health, Shandong University, Jinan, Shandong, China. E-mail: sdweidaimin@163.com

# Table of Contents

**Table of Contents	2**

**Supplementary Figures and Legends	3**

**Supplementary Tables	14**

**List of Abbreviations	22**

**Supplementary Figures and Legends**

**Figure S1. BMP7 and TGM2 expression in placental villi from healthy pregnancies and PE,**

**with supplementary characterization of trophoblast organoid experiments.**

**(A)** RT-qPCR analysis of *BMP7* mRNA expression in placental villous tissues from healthy pregnancy (HP) and preeclampsia (PE) donors. **(B)** Western blot analysis of BMP7 protein expression in placental villous tissues from HP and PE donors. **(C)** RT-qPCR analysis of *TGM2* mRNA expression in placental villous tissues from HP and PE donors. **(D)** Western blot analysis of TGM2 protein expression in placental villous tissues from HP and PE donors. Representative blots and corresponding quantification are shown. Data are represented as mean ± SD. *P* value was calculated by unpaired two-tailed Student’s t-tests and *P*<0.05 was considered statistically significant. ns, not significant. **(E)** Schematic of trophoblast organoid establishment and EVT differentiation. Human trophoblast organoids (TOs) were generated from first-trimester placental villi and maintained in trophoblast organoid medium (TOM) for 3-4 days before switching to EVT medium for 5-10 days. Recombinant TGM2 protein was added at the onset of EVT induction (red dot). **(F)** Representative bright-field images show compact organoids at early stages (Day 1-4) and cellular outgrowths by Day 10. Scale bars: 100 μm.

**Figure S2. ALK2 and ALK3 mediate the regulatory role of BMP7 in trophoblasts.**

**(A)** Primary human trophoblast cells were transfected for 48 hours with 20 nM control nontargeting siRNA, 20 nM siRNA targeting *SMAD1/5/9* (*SMAD1+SMAD5+SMAD9*) or 20 nM siRNA targeting *SMAD4* before treatment with or without 50 ng/mL recombinant BMP7 for 48 hours. The protein levels of the targets were assayed by western blot. **(B)** Primary human trophoblast cells were transfected for 48 hours with 20 nM control nontargeting siRNA or 20 nM siRNA targeting *SMAD2/3* (*SMAD2+SMAD3*) prior to treatment with or without 50 ng/mL recombinant BMP7 for 48 hours. Representative immunoblot images are shown in the left panel. **(C)** Primary human trophoblast cells were pretreated with vehicle (DMSO), the ALK2/ALK3 inhibitor DMH-1 (1 μM), or ALK4, ALK5, or the ALK6 inhibitor SB431542 (10 μM) for 60 minutes followed by treatment with or without BMP7 (50 ng/mL) for 1 hour or 48 hours. The summarized quantitative results for p-SMAD1/5/9 protein levels and TGM2 protein levels are shown. **(D)** Primary human trophoblast cells were transfected with 20 nM non-targeting control siRNA or siRNAs targeting *ALK2* and/or *ALK3* or *ALK4* and *ALK5* for 48 hours before treatment with or without BMP7 (50 ng/mL) for 1 hour (upper panel) or 48 hours (lower panel). Representative immunoblot images are shown in the left panel and the summarized quantitative results for p-SMAD1/5/9 protein levels are shown in the right panel. RT-qPCR was used to examine the *ALK2*, *ALK3*, *ALK4*, and *ALK5* mRNA levels. ns, not significant. **(E)** Immunofluorescence staining for cytokeratin-7 in purified primary human trophoblast cells. Scale bars: 100 μm. Data are represented as mean ± SD. *P* value was calculated by ANOVA (A-D). *P*<0.05 was considered statistically significant. ns, not significant. Columns without letters in common are significantly different (D). These data represent three independent experiments, each carried out in triplicate.

**Figure S3. Single-cell expression and functional association of ALK2 and ALK3 in first-trimester trophoblasts.**

**(A-B)** Violin plot displaying the expression of *ALK2* (A) and *ALK3* (B) in all captured cell types at the first-trimester maternal-fetal interface. High expression of *ALK2* and *ALK3* were detected in VCT and dS. **(C)** Violin plots comparing the expression of *TGM2* and canonical BMP-responsive genes (*ID1* and *ID2*) in ALK2^+^ALK3^+^ VCTs and ALK2^-^ALK3^-^ VCTs. (**D**) Violin plots comparing selected trophoblast function-related genes in ALK2^+^ALK3^+^ VCTs and ALK2^-^ALK3^-^ VCTs. *P*<0.05 was considered statistically significant. ns, not significant. Data are represented as mean ± SD. *P* value was calculated by unpaired two-tailed Student’s t-tests.

**Figure S4. Effects of BMP7 treatment and TGM2 overexpression on proliferation and apoptosis of HTR-8/SVneo and primary human trophoblast cells**

**(A-B)** HTR8/SVneo cells were transfected for 48 hours with a vector plasmid (oe-Ctrl) or TGM2-overexpressing plasmid (oe-*TGM2*) before treatment with or without 50 ng/mL recombinant BMP7. (A) HTR-8/SVneo cell proliferation was examined with EdU assays. Scale bars: 100 μm. (B) Flow cytometry assays were performed to examine the apoptosis rates of HTR-8/SVneo cells. **(C)** Primary human trophoblast cells proliferation after transfection with a vector plasmid (oe-Ctrl) or TGM2-overexpressing plasmid (oe-*TGM2*) for 48 hours before treatment with or without 50 ng/mL BMP7 was examined with EdU assays. Representative images are presented in the left panel, and the summarized quantitative results are presented in the right panel. Scale bars: 100 μm. Data are represented as mean ± SD. *P* value was calculated by ANOVA. *P*<0.05 was considered statistically significant. ns, not significant. These data represent three independent experiments, each carried out in triplicate.

**Figure S5. Loss-of-function analysis of BMP7 and TGM2 in primary human trophoblast cells.**

**(A-B)** Validation of BMP7 knockdown in primary human trophoblast cells by RT-qPCR (A) and western blot (B). **(C-D)** RT-qPCR (C) and western blot (D) analyses of BMP7 and TGM2 expression in primary human trophoblast cells transfected with 20 nM control nontargeting siRNA or 20 nM siRNA targeting *BMP7* for 48 hours, followed by treatment with or without 50 ng/mL recombinant BMP7 for an additional 48 hours. **(E)** Wound-healing assays of primary human trophoblast cells transfected with si-Ctrl or si-*BMP7*, followed by treatment with or without recombinant BMP7. Scale bars: 100 μm. **(F)** Transwell migration and invasion assays of primary human trophoblast cells transfected with si-Ctrl or si-*BMP7*, followed by treatment with or without recombinant BMP7. Scale bars: 100 μm. **(G-H)** Validation of TGM2 knockdown in primary human trophoblast cells by RT-qPCR (G) and western blot (H). **(I-J)** RT-qPCR (I) and western blot (J) analyses of TGM2 expression in primary human trophoblast cells transfected with control siRNA or si-*TGM2*, followed by treatment with or without recombinant BMP7. The four groups were si-Ctrl, si-Ctrl+BMP7, si-*TGM2*, and si-*TGM2*+BMP7. **(K)** Wound-healing assays of primary human trophoblast cells transfected with si-Ctrl or si-*TGM2*, followed by treatment with or without recombinant BMP7. Scale bars: 100 μm. **(L)** Transwell migration and invasion assays of primary human trophoblast cells transfected with si-Ctrl or si-*TGM2*, followed by treatment with or without recombinant BMP7. Scale bars: 100 μm. Data are represented as mean ± SD. P values were determined by t-test or ANOVA, as appropriate. P<0.05 was considered statistically significant. ns, not significant. These data represent three independent experiments, each carried out in triplicate.

**Figure S6. The alterations in renal morphology and function in Ad sFlt-1-induced PE mice.**

**(A)** Body weight at GD0 (the day at which the formation of vaginal plugs was monitored) and GD8 (the day at which the virus was injected). **(B)** PCA plot demonstrating separation between gene expression profiles of the placentas in control (Ad Fc+saline) and PE (Ad sFlt-1+saline) groups (4 placentas *vs*. 5 placentas), as determined by RNA-seq. **(C)** Heatmap depicting the top upregulated DEGs (adjusted *P* value <0.05 and fold change contrast >1.2). **(D)** Histopathological analysis of renal tissue from each group by H&E and PAS staining, and IHC staining for CD34 (a marker of glomerular capillary endothelial injury). **(E)** The summarized quantitative results of signal intensity in IHC staining against CD34 were analyzed with ImageScope. n = 20 glomeruli per mouse. Data are represented as mean ± SD. *P* value was calculated by ANOVA. *P*<0.05 was considered statistically significant. ns, not significant.

**Figure S7. Pharmacological inhibition of TGM2 partially ameliorates PE-like phenotypes in an Ad sFlt-1-induced PE mice model.**

**(A)** Schematic illustration of the animal experimental protocol. Adenoviruses expressing sFlt-1 or control Fc were injected via the tail vein into CD-1 mice on GD8. Cystamine diHCl (50 mg/kg/day) was administered intraperitoneally from GD10 to GD17. Blood pressure was measured and recorded every 2‒3 days from GD7 to GD18. Mice were sacrificed on GD18. N=3. **(B)** Representative images of fetuses and placentas collected from each group at GD18. **(C)** Systolic blood pressure in the indicated groups. **(D)** Fetal weight at GD18. **(E)** Placental weight at GD18. **(F)** Placental efficiency, expressed as fetal/placental weight ratio. Data are represented as mean ± SD. *P* value was calculated by ANOVA. *P*<0.05 was considered statistically significant. ns, not significant.

**Supplementary Tables**

**Table S1. The siRNA sequences used in this study**

| si-RNA |  | Sequence (5’ to 3’) |
| --- | --- | --- |
| si-*ALK3* | Sense | CAGACGUCGUUACAAUCGUTT |
|  | Anti-sense | ACGAUUGUAACGACGUCUGTT |
| si-*ALK4* | Sense | GAAGCCUGGAAUUGCUCAUTT |
|  | Anti-sense | AUGAGCAAUUCCAGGCUUCTT |
| si-*ALK5* | Sense | GGGUCUGUGACUACAACAUTT |
|  | Anti-sense | AUGUUGUAGUCACAGACCCTT |
| si-*BMPR2* | Sense | GGGACAUAAAUCUUGUAAATT |
|  | Anti-sense | UUUACAAGAUUUAUGUCCCTT |
| si-*SMAD1* | Sense | GGUGCUCUAUUGUCUACUATT |
|  | Anti-sense | UAGUAGACAAUAGAGCACCTT |
| si-*SMAD5* | Sense | CCAGCAGUAAAGCGAUUGUTT |
|  | Anti-sense | ACAAUCGCUUUACUGCUGGTT |
| si-*SMAD9* | Sense | CUCUAGUGAAGAAGUUAAATT |
|  | Anti-sense | UUUAACUUCUUCACUAGAGTT |
| si-*SMAD2* | Sense | GGUGUUCGAUAGCAUAUUATT |
|  | Anti-sense | UAAUAUGCUAUCGAACACCTT |
| si-*SMAD3* | Sense | GCGUGAAUCCCUACCACUATT |
|  | Anti-sense | UAGUGGUAGGGAUUCACGCTT |
| si-*SMAD4* | Sense | GCCAGCUACUUACCAUCAUT |
|  | Anti-sense | AUGAUGGUAAGUAGCUGGCTT |
| si-*BMP7* | Sense | ACUCGGCACCCAUGUUCAUTT |
|  | Anti-sense | AUGAACAUGGGUGCCGAGUTT |
| si-*TGM2* | Sense | GCCUGAUCCUUCUAGAUGUTT |
|  | Anti-sense | ACAUCUAGAAGGAUCAGGCTT |
| Negative control | Sense | UUCUCCGAACGUGUCACGUTT |
|  | Anti-sense | ACGUGACACGUUCGGAGAATT |

**Table S2. Specific antibodies used in this study**

| Antibody | Source of species | Catalog number | Application | Manufacturer | Dilution ratio |
| --- | --- | --- | --- | --- | --- |
| Anti-P38 | Rabbit | 4511 | WB | CST | 1:1000 |
| Anti-p-P38 | Rabbit | 8690 | WB | CST | 1:1000 |
| Anti-Erk | Rabbit | 4695 | WB | CST | 1:1000 |
| Anti-p-Erk | Rabbit | 4370 | WB | CST | 1:1000 |
| Anti-JNK | Rabbit | 9252 | WB | CST | 1:1000 |
| Anti-p-JNK | Rabbit | 4668 | WB | CST | 1:1000 |
| Anti-AKT | Rabbit | 9272 | WB | CST | 1:1000 |
| Anti-p-AKT | Rabbit | 9271 | WB | CST | 1:1000 |
| Anti-FAK | Mouse | 66258-1-Ig | WB | Proteintech | 1:2000 |
| Anti-p-FAK | Rabbit | 3281 | WB | CST | 1:1000 |
| Anti-p-SMAD1/5/9 | Rabbit | 13820 | WB | CST | 1:1000 |
| Anti-SMAD1 | Rabbit | 6944 | WB | CST | 1:1000 |
| Anti-P-SMAD2 | Rabbit | 3108 | WB | CST | 1:1000 |
| Anti-SMAD2 | Rabbit | 3103 | WB | CST | 1:1000 |
| Anti-p-SMAD3 | Rabbit | 9520 | WB | CST | 1:1000 |
| Anti-SMAD3 | Rabbit | 9523 | WB | CST | 1:1000 |
| Anti-SMAD4 | Rabbit | 38454 | WB | CST | 1:1000 |
| Anti-β-ACTIN | Mouse | 66009-1-Ig | WB | Proteintech | 1:20000 |
| Anti-TGM2 | Rabbit | HPA021019 | WB | Sigma | 1:2000 |
| Anti-BMP7 | Rabbit | 12221-1-AP | WB/IHC/IF | Proteintech | 1:100 |
| Anti-Cytokeratin 7 | Mouse | 66483-1- Ig | IHC/IF | Proteintech | 1:50 |
| Anti-HLA-G | Mouse | 66447-1-Ig | IHC/IF | Proteintech | 1:50 |
| Anti-Vimentin | Mouse | 60330-1- Ig | IHC/IF | Proteintech | 1:200 |
| Anti-CD34 | Rabbit | A19015 | IHC/IF | ABclonal | 1:50 |
| Anti-CD31 | Rabbit | A19014 | IF | ABclonal | 1:50 |
| Anti-Monocarboxylate Transporter 1 (MCT1) | Chicken | AB1286I | IF | Sigma | 1:100 |
| Anti-Monocarboxylate Transporter 4 (MCT4) | Rabbit | AB3314P | IF | Sigma | 1:200 |
| Anti-rabbit HRP-linked | Goat | 7074 | WB | CST | 1:1000 |
| Anti-mouse HRP-linked | Horse | 7076 | WB | CST | 1:1000 |
| Alexa Fluor 488-conjugated goat anti-rabbit antibody | Goat | A-11070 | IF | Invitrogen | 1:500 |
| Alexa Fluor 594-conjugated goat anti-rabbit antibody | Goat | A-11012 | IF | Invitrogen | 1:500 |
| Alexa Fluor 488-conjugated goat anti-mouse antibody | Goat | A-11001 | IF | Invitrogen | 1:500 |
| Alexa Fluor 488-conjugated goat anti-chicken antibody | Goat | A-11039 | IF | Invitrogen | 1:500 |
| Anti-human  HLA-G | Mouse | 11-499-C100 | IF | Exbio | 1:200 |
| F-actinCoraLite® Plus 488-conjugated Beta Actin Monoclonal antibody | Mouse | CL488-60008 | IF | Proteintech | 1:200 |
| Anti-TGM2 | Rabbit | A21184 | IF | Abclonal | 1:200 |
| Cytokeratin 7 Polyclonal antibody | Rabbit | 15539-1-AP | IF | Proteintech | 1:200 |

**Table S3. Primers used for quantitative PCR**

| Gene | Species |  | Sequence  (5’ → 3’) |
| --- | --- | --- | --- |
| *TGM2* | human | Forward | CAGGAGAAGAGCGAAGGGAC |
|  |  | Reverse | CAAAGGGCGCATCGTACTTG |
| *ALK2* | human | Forward | GTGAAGGTCTCTCCTGCGGTA |
|  |  | Reverse | GCCATCGTTGATGCTCAGTGA |
| *ALK3* | human | Forward | CCTGTTGTCATAGGTCCGTTTT |
|  |  | Reverse | ATCCTGTTCCAAATCACGATTGT |
| *ALK4* | human | Forward | ACTGGTGGCAGAGTTATGAGG |
|  |  | Reverse | GCATACCAACACTCTCGCATC |
| *ALK5* | human | Forward | CAGGACCACTGCAATAAAATAGAACTT |
|  |  | Reverse | TGCCAGTTCAACAGGACCAA |
| *BMPR2* | human | Forward | CACTCAGTCCACCTCATTCATTT |
|  |  | Reverse | TTGTTTACGGTCTCCTGTCAAC |
| *SMAD2* | human | Forward | GCCTTTACAGCTTCTCTGAACAA |
|  |  | Reverse | ATGTGGCAATCCTTTTCGAT |
| *SMAD3* | human | Forward | CCCCAGCACATAATAACTTGG |
|  |  | Reverse | AGGAGATGGAGCACCAGAAG |
| *SMAD4* | human | Forward | CTCATGTGATCTATGCCCGTC |
|  |  | Reverse | AGGTGATACAACTCGTTCGTAGT |
| *ACTB* | human | Forward | CATGTACGTTGCTATCCAGGC |
|  |  | Reverse | CTCCTTAATGTCACGCACGAT |
| *HLA-G* | human | Forward | GAAGAGGAGACACGGAACACC |
|  |  | Reverse | GGTGTGAGAACTGGCCTCG |
| *MMP2* | human | Forward | AGCGAGTGGATGCCGCCTTTAA |
|  |  | Reverse | CATTCCAGGCATCTGCGATGAG |
| *ITGA5* | human | Forward | CGGGCTCCTTCTTCGGATT |
|  |  | Reverse | CACCCCAAGGACAGAGGTAG |
| *MMP14* | human | Forward | CCTTGGACTGTCAGGAATGAGG |
|  |  | Reverse | TTCTCCGTGTCCATCCACTGGT |
| *BMP7* | human | Forward | TAGCCATTTCCTCACCGACG |
|  |  | Reverse | ACGTCTCATTGTCGAAGCGT |
| *Tgm2* | mouse | Forward | CTGGAGAGCAACAAGAGCGA |
|  |  | Reverse | GCTCTTCTCCTGTGGTGTGG |
| *Gapdh* | mouse | Forward | ACTCCACTCACGGCAAATTC |
|  |  | Reverse | TCTCCATGGTGGTGAAGACA |

**Table S4. Baseline characteristics of the participants for IF staining, RT-qPCR and western blot.**

| Characteristics | PE group (n=6) | HP group (n=6) | *P* value |
| --- | --- | --- | --- |
| Maternal age (years) | 33.67±2.58 | 34.83±3.31 | 0.512 |
| Body mass index (kg/m^2^) | 35.10±6.82 | 29.76±2.76 | 0.122 |
| Parity≥1 | 4 (66.67%) | 5 (83.3%) | 1.00 |
| Systolic pressure (mmHg) | 153.33±11.08 | 110.50±13.28 | <0.001 |
| Diastolic pressure (mmHg) | 104.33±5.16 | 76.67±8.52 | <0.001 |
| Blood test results |  |  |  |
| Platelets (10^9^/L) | 205.83±39.09 | 206.33±54.04 | 0.986 |
| ALT (U/L) | 13.00 (6.75, 16.00) | 8.00 (6.75, 33.75) | 0.485 |
| AST (U/L) | 23.50 (18.00, 25.25) | 17.00 (17.00, 28.00) | 0.310 |
| Gestational weeks at birth (days) | 253.00±22.79 | 269.33±5.09 | 0.142 |
| Birthweight of newborns (g) | 2675.00±958.66 | 3501.67±387.27 | 0.094 |

Data are presented as the mean ± standard deviation, median (interquartile range) or n (%). For normally distributed data, the mean ± standard deviation is presented. Otherwise, the median (interquartile range) is presented. *P*<0.05 was considered statistically significant. Abbreviations: ALT, alanine transaminase; AST, aspartate transaminase; HP, healthy pregnancy; IF, immunostaining; PE, preeclampsia.

**Table S5. Clinical and laboratory variables of the participants for ELISA.**

| Characteristics | PE group (n=23) | HP group (n=11) | *P* value |
| --- | --- | --- | --- |
| Maternal age (years) | 32.83±6.162 | 33.73±3.467 | 0.656 |
| Body mass index (kg/m^2^) | 30.48 (28.75,34.02) | 27.18 (25.71, 30.67) | 0.042 |
| Parity≥1 | 13 (61.9%) | 8 (38.1%) | 0.465 |
| Systolic pressure (mmHg) | 156 (146,168) | 120 (115,125) | <0.001 |
| Diastolic pressure (mmHg) | 101.57±11.18 | 74.27±9.18 | <0.001 |
| Blood test results |  |  |  |
| Hemoglobin (g/L) | 114.65±10.24 | 113.27±5.88 | 0.623 |
| Platelets (10^9^/L) | 206.35±54.63 | 193.36±32.48 | 0.394 |
| ALT (U/L) | 15.93±6.55 | 9.75±3.64 | 0.007 |
| AST (U/L) | 23.96±9.01 | 14.66±3.01 | <0.001 |
| Albumin (g/L) | 33.75±4.06 | 35.87±1.47 | 0.034 |
| Prothrombin time (PT, s) | 12.1 (11.5,12.7) | 9.9 (9.5,11.1) | 0.003 |
| APTT (s) | 30.56±2.84 | 27.22±1.84 | 0.001 |
| Fibrinogen (g/L) | 4.27±1.10 | 4.19±0.85 | 0.820 |
| Gestational weeks at birth (days) | 254 (219,261) | 271 (266,281) | 0.02 |
| Birthweight of newborns (g) | 2247.17±745.18 | 3031.82±605.74 | 0.005 |

Data are presented as the mean ± standard deviation, median (interquartile range) or n (%). For normally distributed data, the mean ± standard deviation is presented. Otherwise, the median (interquartile range) is presented. *P*<0.05 was considered statistically significant. Abbreviations: ALT, alanine transaminase; APTT, activated partial thromboplastin time; AST, aspartate transaminase, HP, healthy pregnancy; PE, preeclampsia; PT, prothrombin time.

**List of Abbreviations**

ALK, activin receptor-like kinase

ANOVA, analyses of variance

BMPs, bone morphogenetic proteins

BSA, bovine serum albumin

Cystamine dihydrochloride, Cystamine diHCl

CVD, cardiovascular diseases

DAB, diaminobenzidine

DAPI, 4′,6-diamidino-2-phenylindole

DEG, differentially expressed gene

ELISA, enzyme-linked immunosorbent assay

EVT, extravillous trophoblast

FBS, fetal bovine serum

GD, gestation day

GO, Gene Ontology

H&E, hematoxylin and eosin

IF, immunofluorescence

IgG, immunoglobulin G

IHC, immunohistochemistry

KEGG, Kyoto Encyclopedia of Genes and Genomes

mRNA, messenger RNA

PAS, periodic acid–Schiff

PBS, phosphate-buffered saline

PCA, principal component analysis

PE, preeclampsia

PSM, propensity score matching

PVDF, polyvinylidene difluoride

RT‒qPCR, quantitative real-time reverse-transcription

SBP, systolic blood pressure

SDS-PAGE, sodium dodecyl sulfate‒polyacrylamide gel electrophoresis

sFlt-1, soluble fms-like tyrosine kinase–1

siRNA, small interfering RNA

STB, syncytiotrophoblasts

TBS, Tris-buffered saline

TGF-β, transforming growth factor-β

TGM2, tissue transglutaminase

TO, trophoblast organoids

TOM, trophoblast organoid medium

VCT, villous cytotrophoblast
